# A Shh/Gli-driven three-node timer motif controls temporal identity and fate of neural stem cells

**DOI:** 10.1126/sciadv.aba8196

**Published:** 2020-09-16

**Authors:** José M. Dias, Zhanna Alekseenko, Ashwini Jeggari, Marcelo Boareto, Jannik Vollmer, Mariya Kozhevnikova, Hui Wang, Michael P. Matise, Andrey Alexeyenko, Dagmar Iber, Johan Ericson

**Affiliations:** 1 Department of Cell and Molecular Biology, Karolinska Institutet, S-171 77 Stockholm, Sweden.; 2 D-BSSE, ETF Zürich, Mattenstrasse 26, 4058 Basel, Switzerland.; 3 Swiss Institute of Bioinformatics (SIB), Mattenstrasse 26, 4058 Basel, Switzerland.; 4 Department of Neuroscience and Cell Biology, Rutgers-Robert Wood Johnson Medical School, 675 Hoes Lane, Piscataway, NJ, 08854, USA.; 5 Department of Microbiology, Tumor and Cell Biology, Karolinska Institutet, Stockholm, Sweden.; 6 Science for Life Laboratory, Box 1031, 17121, Solna, Sweden.

## Abstract

How time is measured by neural stem cells during temporal neurogenesis has remained unresolved. By combining experiments and computational modeling, we define a Shh/Gli-driven three-node timer underlying the sequential generation of motor neurons (MNs) and serotonergic neurons in the brainstem. The timer is founded on temporal decline of Gli-activator and Gli-repressor activities established through down-regulation of Gli transcription. The circuitry conforms an incoherent feed-forward loop, whereby Gli proteins not only promote expression of Phox2b and thereby MN-fate but also account for a delayed activation of a self-promoting transforming growth factor–β (Tgfβ) node triggering a fate switch by repressing Phox2b. Hysteresis and spatial averaging by diffusion of Tgfβ counteract noise and increase temporal accuracy at the population level, providing a functional rationale for the intrinsically programmed activation of extrinsic switch signals in temporal patterning. Our study defines how time is reliably encoded during the sequential specification of neurons.

## Teaser

A Shh/Gli-driven incoherent feed-forward loop circuit encodes time and counterbalances noise in temporal neural patterning.

## INTRODUCTION

Time is a central axis of information during embryogenesis, but few mechanisms explaining the timing of developmental events have been resolved (*1*–*4*). In the forming central nervous system (CNS), defined pools of multipotent neural stem cells (NSCs) produce distinct cell types in a specific sequential order and over defined time frames. In this process, aging NSCs become progressively restricted in their developmental potential by losing competence to generate early-born cell types (*5*), and genome-wide analyses have revealed that NSCs undergo dynamic transcriptional changes over time (*6*). However, the composition and functional properties of time-encoding circuitries determining time frames and point of transitions have not been resolved in any model system (*7*, *8*). Temporal neural patterning in vertebrates is a slow process progressing over days or even weeks depending on species (*9*, *10*). Yet, in several lineages, progenitors undergo coordinated and fast temporal transitions (*9*–*13*). Biological timers regulating temporal neurogenesis are therefore likely to exhibit properties that counterbalance noise in regulatory networks, but how this is achieved at the molecular level remains unknown.

Temporal patterning contributes to the generation of neural cell diversity at all axial levels of the CNS, but only few transcription factors (TFs) and/or signaling molecules regulating temporal fate and potency have been defined in various temporal lineages of the vertebrate CNS (*11*, *13*). To approach the question of time, we focused on a relatively well-defined lineage in the ventral brainstem that sequentially produces motor neurons (MNs), serotonergic neurons (5HTNs), and oligodendrocyte precursors (OPCs) (*12*, *14*). The lineage is induced by Sonic hedgehog (Shh) and defined by the expression of the TF Nkx2.2 (*12*), and the temporal progression of differentiation is easy to monitor as the NSC pool remains at a fixed position and does not comprise the specification of proliferative intermediate progenitors as in the developing neocortex (*13*). Nkx2.2^+^ NSCs show, otherwise, many common features to cortical progenitors, genetic lineage-tracing studies support that young NSCs are multipotent and competent to generate early- and late-born neurons, and aging NSCs become progressively restricted in their potential over time (*12*, *13*, *15*, *16*). In addition, the transition from early to late phases of neurogenesis is governed by late-acting extrinsic signals whose activation is intrinsically programmed within the lineage (*15*, *17*, *18*). Young Nkx2.2^+^ NSCs coexpress early- and late-acting fate determinants (*12*, *15*, *19*–*21*), but the activity of the TF Phox2b predominates by specifying MN fate (*15*, *21*). Once Phox2b is down-regulated or genetically ablated, MN production is terminated and 5HTNs are generated by default (*12*), suggesting that Phox2b functions as temporal effector output. Activators of Phox2b have not been defined, but a self-sustained and temporally delayed activation of Tgfβ operates as an extrinsic signal that triggers MN-to-5HTN fate switch by repressing Phox2b (*15*). Thus, Phox2b and Tgfβ are important regulatory components of a timer circuitry, but to understand how time is set by the network, it is necessary to define activators of Phox2b and resolve how the temporally gated activation of Tgfβ is mechanistically implemented. Here, we define a Shh/Gli-driven three-node circuitry, which explains how time is encoded in the Nkx2.2^+^ lineage and provide evidence that intrinsically programmed activation of extracellular switch signals fulfill an important role to counterbalance noise through spatial averaging, which is unattainable with temporal networks based exclusively on intrinsic transcriptional regulators.

## RESULTS

### Shh/Gli signaling promotes expression of Phox2b

**Fig. 1:**
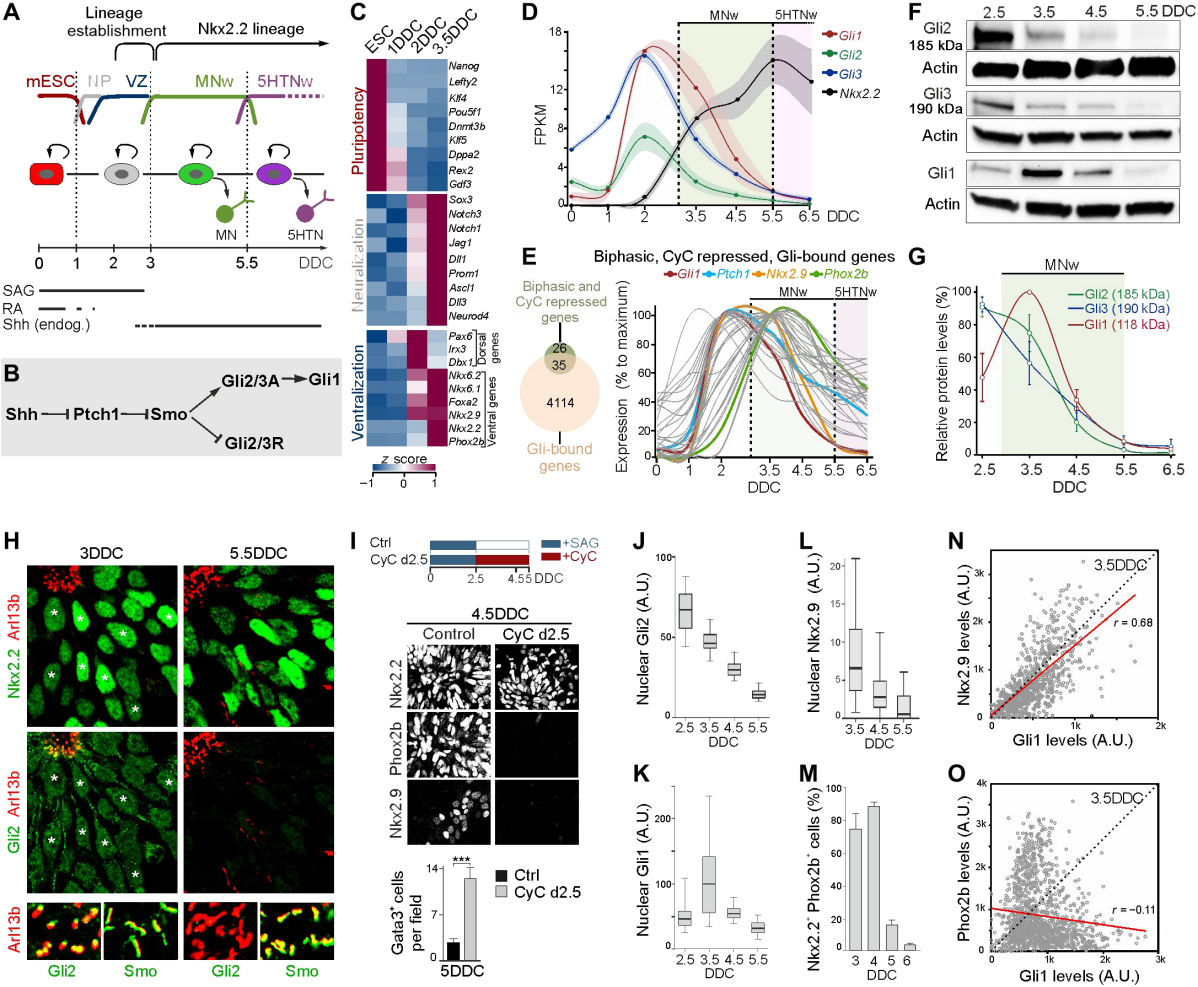
Gli proteins are temporal regulators of Phox2b. (**A**) Scheme of mESC differentiation. (**B**) Scheme of Shh signaling pathway. (**C**) Heatmap of expression levels of genes associated with pluripotency, neuralization, and ventralization. (**D**) Gene expression levels fragments per kilobase of transcript per million mapped read (FPKM) in NSCs over time. Shaded area, SEM (**E**) Genes with biphasic expression profile, repressed at 3.5DDC by CyC treatment (0DDC to 3.5DDC) and bound by Gli1 or Gli3, and their relative temporal expression profile. (**F** and **G**) Western blot for Gli1-3 proteins in NSCs at different DDC and corresponding quantification. (**H**) Immunofluorescence of Arl13b, Nkx2.2, Gli2, and Smo at 3DDC and 5.5DDC. (**I**) Effect of treatment of cultures with CyC from 2.5DDC on Nkx2.2, Phox2b, and Nkx2.9 expression at 4.5DDC and number of Gata3^+^ 5HTNs at 5DDC. (**J** to **L**) Box plot of Gli2, Gli1, and Nkx2.9 expression levels in Nkx2.2^+^ nuclei at different DDC. Whiskers, 5th to 95th percentile; outliers were omitted. (**M**) Quantification of Nkx2.2^+^ NSCs expressing Phox2b over time. (**N** and **O**) Scatter plot of nuclear protein levels of Gli1 with Nkx2.9 or Phox2b in Nkx2.2^+^ nuclei at 3.5DDC. Trend line in red; *r*, correlation coefficient. (F, I, and M) Error bars, means ± SEM; asterisks, Student’s *t* test, ***P ≤ 0.001. MNw, motor-neuron window; 5HTNw, serotonergic-neuron window; A.U., arbitrary units.

The sequential specification of MNs, 5HTNs, and OPCs by Nkx2.2^+^ NSCs is recapitulated in mouse embryonic stem cell (ESC) cultures in response to timed activation of Shh and retinoic acid signaling (*15*). To define genome-wide transcriptional changes over time, we determined the transcriptome of Nkx2.2^+^ NSCs isolated at different time points by RNA sequencing (RNA-seq). In this differentiation paradigm, >90% of Sox3^+^ NSCs express Nkx2.2 at 3.5 days in differentiation conditions (DDC) (*15*). Since the temporal patterning process is slow and progresses over days (*15*), cells isolated at a given time were examined at the population level to even out changes of gene expression associated with cell cycle progression and neuronal differentiation (*22*). We hypothesized that activators driving *Phox2b* are progressively lost over time since Phox2b becomes down-regulated and cells undergo a MN-to-5HTN fate switch in the absence of Tgfβ signaling, but on a delayed temporal schedule (*15*). Therefore, we determined genes down-regulated between 3.5DDC, when Phox2b expression and MN production has been initiated, and 5.5DDC when Phox2b becomes down-regulated in NSCs (Fig. 1A) (*15*). Approximately 2200 genes showed a significant decline over this time frame [*P* ≤ 0.05; log_2_ fold change (FC) ≥ 0.22]. The number of genes was markedly reduced with increased FC stringency, and only 27 genes remained at a FC of ~5.6 [log_2_(FC) ≥ 2.5] (fig. S1A). Among these were *Gli1*, *Gli2*, and *Gli3* (fig. S1A), which encode zinc-finger TFs that transduce Shh signaling within the nuclei (*23*).

Gli2 and Gli3 are bifunctional TFs that are processed into repressors (GliR) in the absence of Shh but stabilized as full-length activators (GliA) within cilia by Smo (Smoothened) in response to binding of Shh to Ptch1 (Fig. 1B) (*23*). Gli1, in turn, is an obligate activator and a direct target of the Shh pathway (Fig. 1B) and is together with Ptch1 commonly used as an indicator of ongoing Shh signaling (*24*). *Gli1* and *Ptch1* exhibited a biphasic temporal expression: they were up-regulated during ventralization of NSCs and induction of the Nkx2.2^+^ lineage (~1DDC to 3DDC) followed by a progressive decline over the Phox2b^+^ MN window (3DDC to 5.5DDC) (Fig. 1, C to E). *Gli2* and *Gli3* showed similar biphasic behavior but were expressed at low levels at ESC stages (Fig. 1D). We identified 61 genes with biphasic expression similar to *Gli1* and whose induction was inhibited by early treatment of cells with the Smo antagonist cyclopamine (CyC) (Fig. 1E and table S1). Gli1-3 bound (*25*, *26*) in proximity to ~57% (35) of these genes (Fig. 1E and table S1), including *Phox2b* and *Nkx2.9*, which encode a Shh-regulated TF transiently expressed by Nkx2.2^+^ NSCs (Fig. 1E) (*27*). This identifies Gli1-3 as putative activators of Phox2b expression.

Biochemical analyses showed that the down-regulation of *Gli* transcription was translated into a progressive decay of full-length activator forms of Gli2 (Gli2A, 185 kDa) and Gli3 (Gli3A, 190 kDa) between 2.5DDC and 5.5DDC (Fig. 1, F and G). Gli1 showed a similar profile but reached peak values at 3.5DDC, consistent with the notion that Gli1 is a target of Gli2A/Gli3A (Fig. 1, F and G). Gli1-3 proteins were detected in nuclei and Arl13b^+^ cilia at 3DDC but not at 5.5DDC (Fig. 1H and fig. S1, C and D). Smo translocates to cilia in response to binding of Shh to Ptch1 (*23*) and localized to Arl13b^+^cilia between 3DDC and 5.5DDC (Fig. 1H). Thus, down-regulation of *Gli* transcription mediates a progressive desensitization of the Shh pathway through loss of GliA, despite that Shh/Smo signaling is active over this period. The progressive loss of Gli1-3 expression correlated with the down-regulation of Phox2b and Nkx2.9 (fig. S1B). Inhibition of Shh/Smo signaling by CyC subsequent to the establishment of the Nkx2.2^+^ lineage resulted in premature down-regulation of Phox2b and Nkx2.9 in Nkx2.2^+^ NSCs and increased generation of Gata3^+^ 5HTNs (Fig. 1I). Expression of Nkx2.2 was not affected by CyC treatment initiated at 2.5DDC (Fig. 1I) consistent with data suggesting that induction but not maintenance of Nkx2.2 expression relies on Shh signaling (*28*). These data show that Shh/Gli signaling is required for sustained expression of Phox2b and Nkx2.9 and suggest that inhibition of Shh signaling is sufficient to trigger a MN-to-5HTN fate switch.

Despite the overall decline of Gli proteins over time, there was notable fluctuations of Gli1 and Gli2 expression levels between individual Nkx2.2^+^ nuclei at a given time examined (Fig. 1, J and K). Gli1 is a readout of Shh signaling activity (*29*), and there was a direct correlation between Nkx2.9 and Gli1 expression levels at 3.5DDC (Fig. 1N). Moreover, the expression of Nkx2.9 declined over time in a manner similar to Gli proteins (Fig. 1, J to L, and fig. S1B), suggesting a GliA dose-dependent regulation of Nkx2.9. In contrast, there was no correlation between Phox2b and Gli1 expression levels (Fig. 1O), implying that regulators, in addition to GliA, are likely to influence Phox2b expression. Nevertheless, the fraction of Nkx2.2^+^ NSCs expressing Phox2b remained largely constant between 3DDC and 4.5DDC and only dropped markedly at 5DCC to 6DDC when Gli expression approached undetectable levels (Fig. 1, J, K, and M, and fig. S1B) implying that the GliA level sufficient to sustain *Phox2b* transcription is very low.

### Ptch1-independent establishment of parallel temporal GliA and GliR gradients

**Fig. 2:**
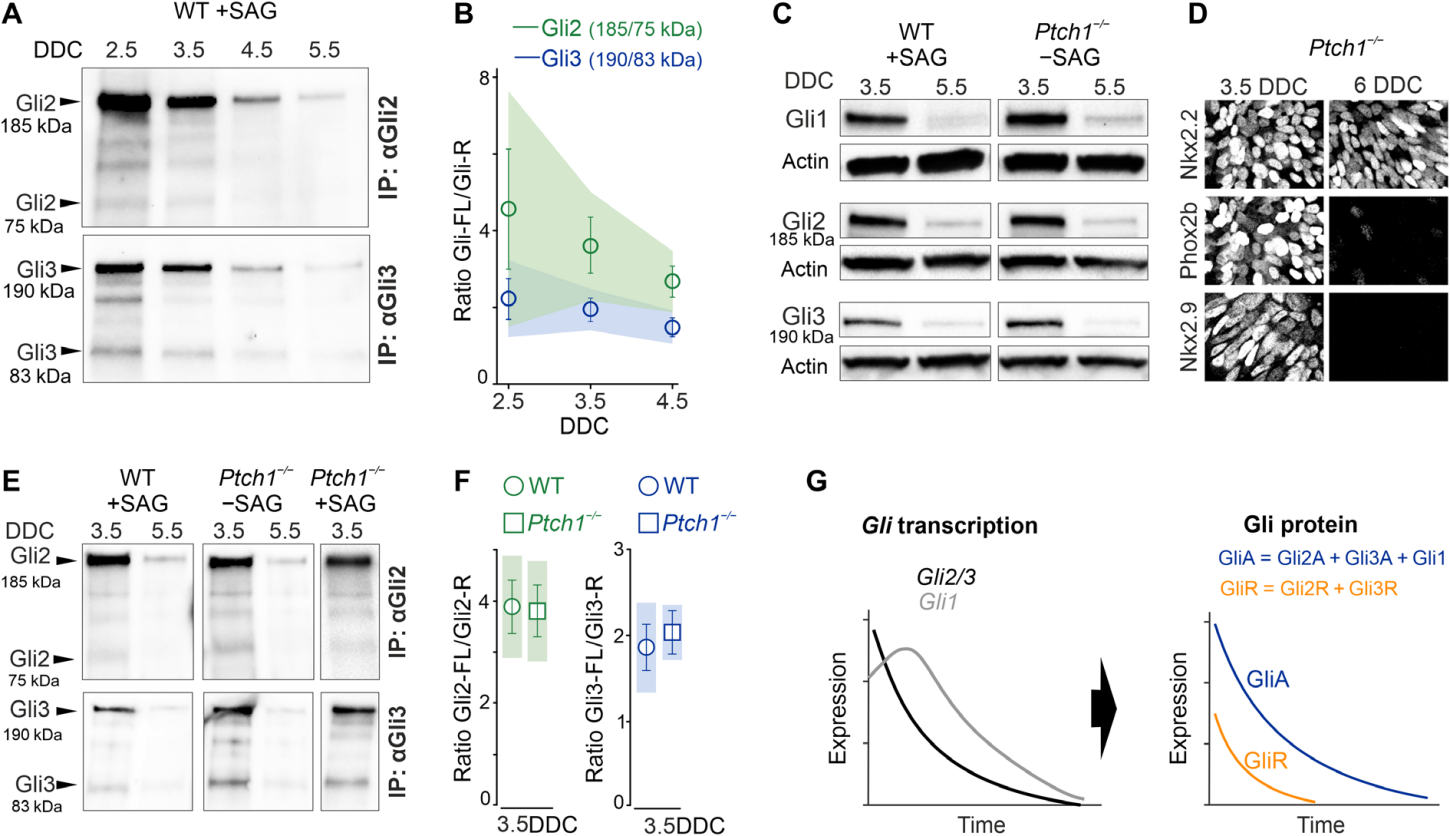
Down-regulation of *Gli* genes establishes parallel temporal GliA/GliR gradients. (**A** and **B**) Western blot of immunoprecipitated (IP) Gli2 or Gli3 protein from NSCs isolated at different DDC and quantification of protein ratios. (**C**) Western blot of Gli1-3 in WT and *Ptch1^−/−^* NSCs at 3.5DDC and 5.5DDC. (**D**) Immunofluorescence of Nkx2.2, Phox2b, and Nkx2.9 in *Ptch1*^−/−^ ESC cultures at 3.5DDC and 6DDC. (**E** and **F**) Western blot of IP Gli2 or Gli3 protein from WT or *Ptch1*^−/−^ NSCs isolated at 3.5DDC and 5.5DDC and differentiated in the presence or absence of SAG as indicated. Quantification of protein ratios of Gli2 and Gli3 bands in WT (+SAG) and *Ptch1^−/−^*(−SAG) NSCs at 3.5DDC. (**G**) Down-regulation of *Gli* genes produces parallel temporal GliA and GliR gradients. (B and F) Error bars, means ± SEM; shaded area, 95% confidence intervals (CIs).

Immunoprecipitation assays revealed the presence of processed repressor forms of Gli2 (75 kDa; Gli2R) and Gli3 (83 kDa; Gli3R), which declined similarly to their respective full-length activator forms between 2.5DDC and 5.5DDC (Fig. 2A). Gli3A/Gli3R ratios remained largely constant, while Gli2A/Gli2R ratios decreased somewhat over time (Fig. 2B). Ptch1 is up-regulated in response to Shh signaling, in which feedback can inhibit the Shh pathway, as free Ptch1 promotes GliR formation through Smo inhibition (*23*). Analysis of differentiating *Ptch1^−/−^* ESCs showed that the temporal expression profiles of *Gli* genes, Gli1-3 activator proteins, Nkx2.2, Nkx2.9, and Phox2b, and induction of *Tgf*β*2* at 6.5DDC were similar in *Ptch1*^**−/−**^ and wild-type (WT) ESC cultures (Fig. 2, C and D, and figs. S1B and S2, A and D). These data establish that Ptch1 function is largely dispensable in the temporal differentiation process. Gli2R and Gli3R were detected in *Ptch1*^−/−^ cultures and at GliA/GliR ratios similar to controls (Fig. 2, E and F). *Ptch2* (*30*) was expressed at extremely low levels in control and *Ptch1^−/−^* cells (fig. S2, B and C). Gli2R and Gli3R were present also in *Ptch1*^**−/−**^ ESC cultures treated with Shh Ag 1.3 (SAG) (Fig. 2E), which activates Smo downstream of Ptch1 and Ptch2 (*30*). These data reveal that a fraction of bifunctional Gli proteins are processed into GliR forms in conditions of fully activated Smo and independent of Ptch1-mediated feedback. The amount of processed Gli2R and Gli3R as well as full-length Gli2 and Gli3 produced at a given time is instead determined by the level of *Gli2, Gli3* transcription, respectively (fig. S3). Thus, down-regulation of *Gli* genes produces parallel declining GliA and GliR gradients in the lineage (Fig. 2G).

### The temporal network conforms a three-node incoherent feed-forward loop circuitry

**Fig. 3:**
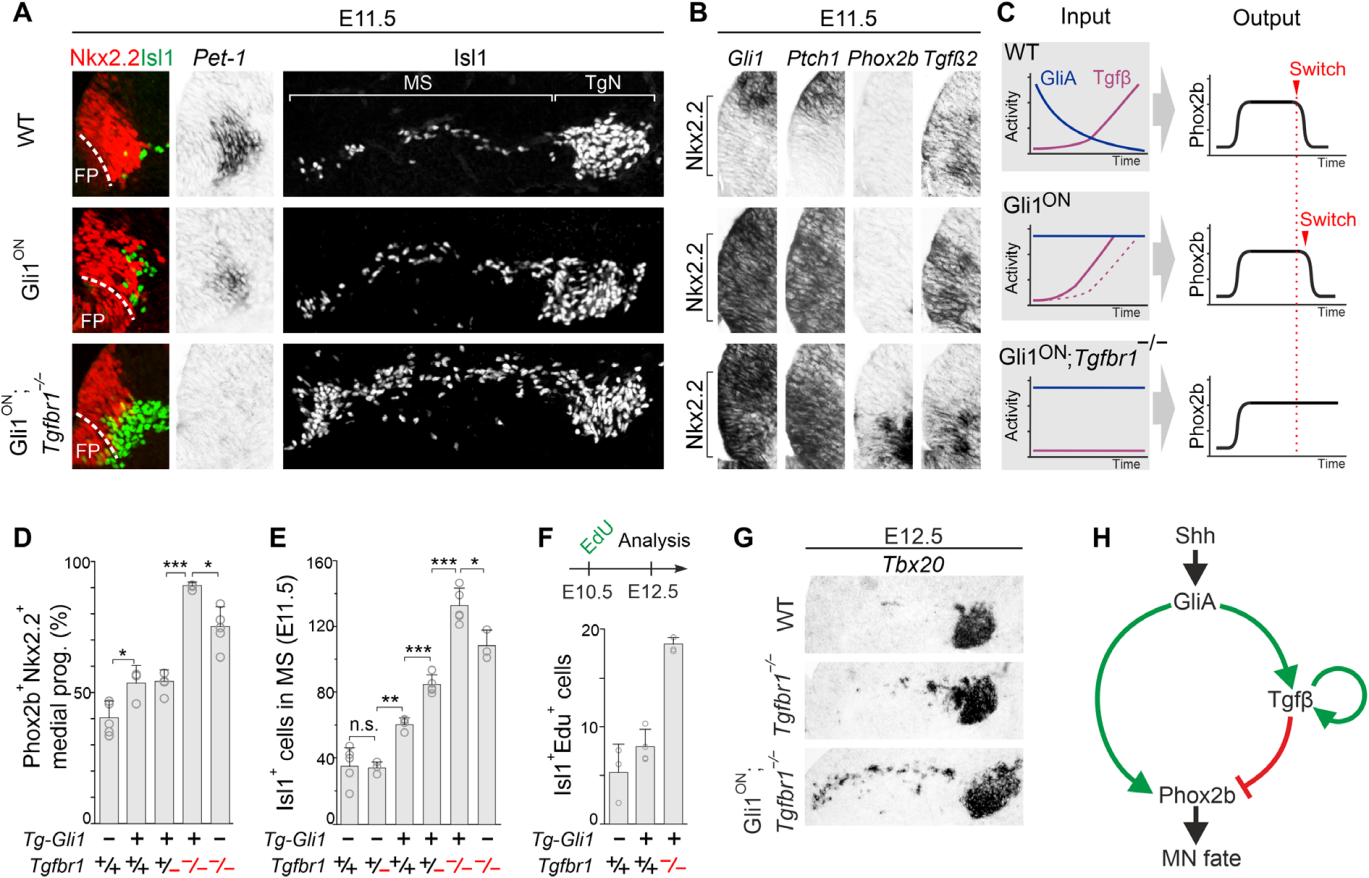
A GliA-driven IFFL network controls the MN-to-5HTN switch time. (**A**, **B**, and **G**) Transverse sections at r2/3 ventral hindbrain level of mouse embryos. (A and B) Expression of Nkx2.2, Isl1, *Pet1*, *Gli1*, *Ptch1*, *Phox2b*, and *Tgf*β*2* at E11.5 in WT and mutant embryos. (**C**) Summary of the effects on GliA, Tgfβ, and Phox2b expression in different mouse mutants. (**D**) Quantification per hemisection of Nkx2.2^+^Phox2b^+^NSCs at E10.5 in WT and mutant backgrounds. Data from WT, *Tgfbr1^−/−^*: *n* = 5; *Tg-Gli1^+^::Tgfbr1^+/−^*: *n =* 4; *Tg-Gli1^+^::Tgfbr1^+/+^*, *Tg-Gli1^+^::Tgfbr1^−/−^*: *n =* 3 animals per group. (**E**) Quantification per hemisection of Isl1^+^-MNs at E11.5 in WT and mutant backgrounds. Data from WT, *Tg-Gli1^+^::Tgfbr1^+/−^*, *Tg-Gli1^+^::Tgfbr1^−/−^*: *n =* 5; *Tg-Gli1^+^::Tgfbr1^+/+^*: *n =* 4; *Tg-Gli1^−^::Tgfbr1^+/−^*, *Tg-Gli1^−^::Tgfbr1^−/−^*: *n =* 3 animals per group. (**F**) Quantification of Isl1^+^Edu^+^ cells at E12.5 in WT (*n =* 3), Gli1^ON^ (*n =* 4), and Gli1^ON^:*Tgfbr1*^mut^ (*n* = 3) embryos pulsed with EdU at E10.5. (G) Expression of *Tbx20*^+^-MNs at E12.5 in WT, *Tgfbr1*^mut^, and Gli1^ON^:*Tgfbr1*^mut^ embryos. (**H**) Schematic of the IFFL network. (D to F) Error bars, means ± SD. Asterisks, Student’s *t* test, *P ≤ 0.05, **P ≤ 0.01, and ***P ≤ 0.001. FP, floor plate; MS, migratory stream; TgN, trigeminal nuclei.

To define the effect of constant GliA input on temporal output, we generated mice in which Gli1 was constitutively expressed in the Nkx2.2^+^ lineage by crossing a *ROSA26-Gli1^FLAG^* transgene (*31*) with a *Nkx6.2-Cre* mouse line (*15*) (hereafter termed Gli1^ON^ mice) (fig. S4A). *Ptch1* is down-regulated in Nkx2.2^+^ NSCs by embryonic day 11.5 (E11.5) but was sustained at high levels in Gli1^ON^ mice over this period (Fig. 3B), consistent with continuous GliA expression at high levels. Forced Gli1 expression did not result in any overt spatial patterning phenotype (Fig. 3A and fig. S4B), and the number of Isl1^+^ trigeminal MNs was similar to controls at E10.5 (fig. S4A), a time when most MNs have been specified (*12*). In controls at E11.5, MN production is terminated, and the generation of *Pet1*^+^ 5HTNs is initiated (Fig. 3A) (*12*). Most sl1^+^MNs have migrated laterally to form the trigeminal nuclei but some late-born MNs are still present in the migratory stream (MS) (Fig. 3A), and *Phox2b*, *Ptch1*, and *Gli1* have been down-regulated and *Tgf*β*2* up-regulated in Nkx2.2^+^ NSCs (Fig. 3B). There was a surplus of Isl1^+^MNs in the MS and reduction of *Pet1*^+^ 5HTNs in Gli1^ON^ mice at E11.5 (Fig. 3A), but the expression of *Phox2b* in Nkx2.2^+^ NSCs was repressed (Fig. 3B). This suggests a mild temporal extension of MN production in Gli1^ON^ mice but underpins that *Phox2b* is suppressed and cells undergo a MN-to-5HTN fate switch on an almost normal temporal schedule in conditions of constant GliA input (Fig. 3C).

The mild temporal phenotype in Gli1^ON^ mice was associated with a marked up-regulation of *Tgf*β*2* at E11.5 (Fig. 3B), revealing a feed-forward propagation of *Tgf*β*2* transcription by GliA. This implies a three-node circuitry forming an incoherent feed-forward loop (IFFL), whereby GliA activates not only Phox2b but also the suppressive Tgfβ node negatively regulating Phox2b (Fig. 3H). This predicts that the MN window should be extended if GliA is maintained and Tgfβ concurrently inactivated, and to test this, we crossed Gli1^ON^ mice onto a *Tgfbr1* mutant background. Removal of one or both copies of *Tgfbr1* resulted in progressively more pronounced temporal phenotypes, as determined by quantification of Phox2b^+^ NSCs and Isl1^+^ MNs and by 5-Ethynyl-2’-deoxyuridine (EdU)–birth dating experiments (Fig. 3, D to F, and fig. S5A). In Gli1^ON^*:Tgfbr1*^**−/−**^ at E11.5, there was a massive accumulation of premigratory and migrating MNs, a complete lack of *Pet1*^+^ 5HTNs and maintained expression *Phox2b* in NSCs (Fig. 3, A and B). The extension of MN production was more pronounced in Gli1^ON^*:Tgfbr1*^**−/−**^ as compared to *Tgfbr1*^**−/−**^ mutants (Fig. 3, E and G, and fig. S5B), supporting that the delayed fate switch in *Tgfbr1* mutants (*15*) occurs due to depletion of GliA. In addition, *Tgf*β*2* expression was reduced in Gli1^ON^*:Tgfbr1*^**−/−**^ relative to Gli1^ON^ mice (Fig. 3B), suggesting that positive feedback signaling by Tgfβ is necessary for robust induction of *Tgf*β*2* downstream of GliA (Fig. 3H). Collectively, these data support an IFFL regulatory topology for the GliA-Phox2b-Tgfβ motif (Fig. 3H) and establish that Tgfβ predominates over the Shh pathway by suppressing Phox2b even if cells express GliA at levels sufficient to sustain *Phox2b* transcription.

### Different GliR sensitivities account for sequential activation of *Phox2b* and *Tgf*β*2*

**Fig. 4:**
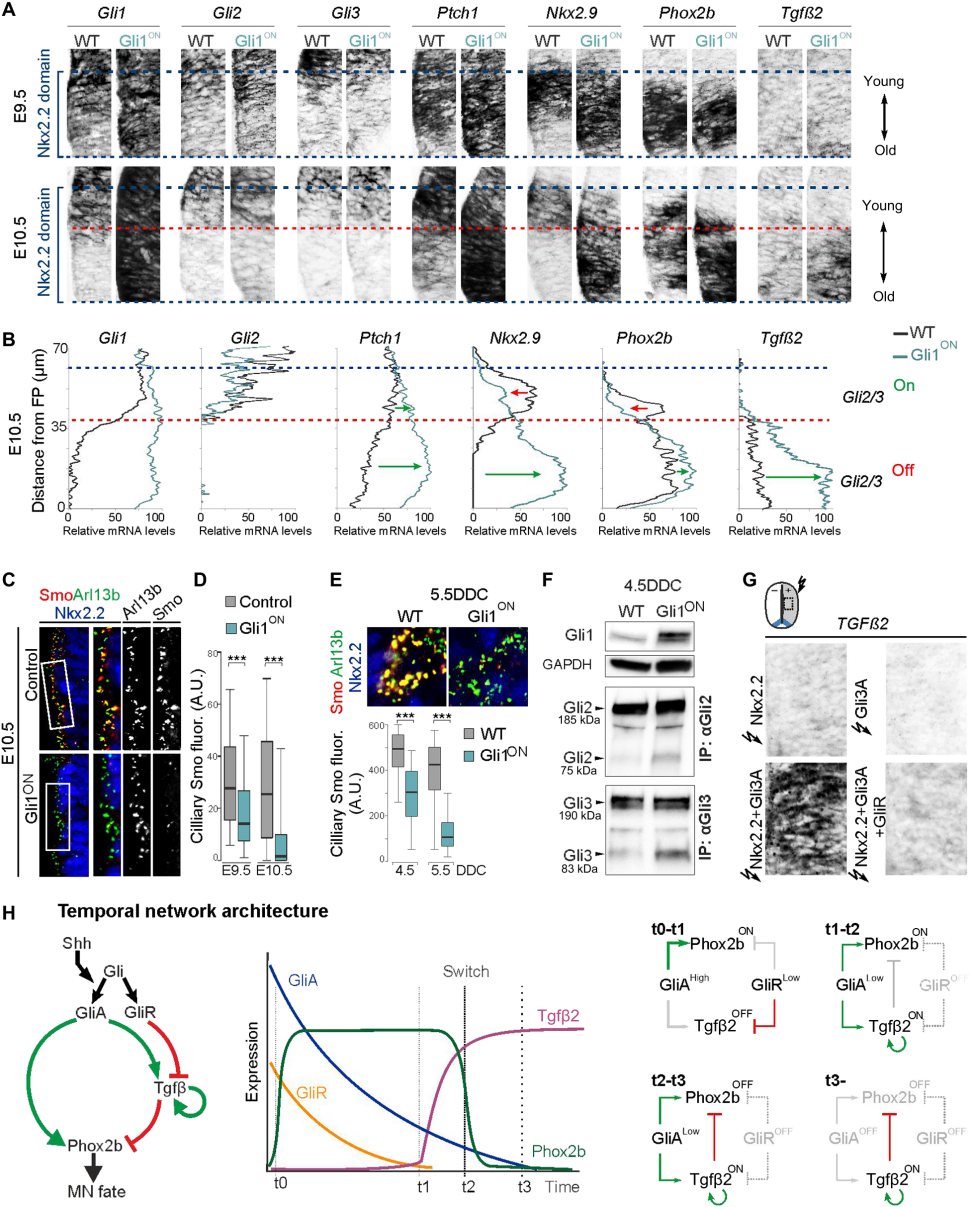
Differential regulation of gene expression by GliA and GliR levels. (**A** and **C**) Transverse sections at r2/3 ventral hindbrain level of mouse embryos. (A and **B**) Expression of *Gli1-3*, *Ptch1*, *Nkx2.9*, *Phox2b*, and *Tgf*β*2* in WT and Gli1^ON^ embryos at E9.5 and E10.5 in Nkx2.2 progenitor domain (A) and plots of relative transcript expression levels along the ventral-dorsal extent of the Nkx2.2 progenitor domain at E10.5 (B). Blue dashed lines delimit Nkx2.2 domain; red dashed line, approximate ventral limit of *Gli2/Gli3* expression. (C and **D**) Expression of Smo in Arl13b^+^ cilia within Nkx2.2 domain at E10.5 and box plots of Smo protein levels in individual Arl13^+^ cilia at E9.5 and E10.5 in WT and Gli1^ON^ embryos. (**E**) Expression of Smo, Arl13b, and Nkx2.2 in WT and Gli1^ON^ ESC cultures at 5.5DDC and box plots of Smo levels in Arl13^+^ cilia at 4.5DDC and 5.5DDC. (**F**) Western blot of Gli1 and of IP Gli2 or Gli3 proteins from WT and Gli1^ON^ NSCs isolated at 4.5DDC. (**G**) Electroporation of CAGG constructs expressing Nkx2.2, Gli3A, Nkx2.2 + Gli3A, Nkx2.2 + Gli3A + GliR at HH12 and analysis of *TGF*β*2* expression in the neural tube (boxed region) after 40 hours. (**H**) Summary of the temporal network. (D and E) Box plots, whiskers define 5th to 95th percentile, outliers are omitted. Asterisks, pairwise Wilcoxon test, ***P ≤ 0.001.

GliA promotes both *Phox2b* and *Tgf*β*2*, raising the key question how *Tgf*β*2* induction can be circumvented at early stages when GliA activity peaks? As the down-regulation of *Gli* genes produces parallel declining GliA and GliR temporal gradients (Fig. 2G), we considered that the delayed activation of *Tgf*β*2* could be explained by an inhibitor-titration regulation, a regulatory motif known to convey nonlinear switch-like responses (*32*, *33*). To explore this possibility, we characterized the spatiotemporal dynamics of gene expression in Nkx2.2^+^ NSCs in vivo by semiquantitative in situ hybridization and quantitative polymerase chain reaction (qPCR). Induction of the Nkx2.2^+^ domain and subsequent MN-to-5HTN fate switch progress in a ventral-to-dorsal (V → D) manner (*12*, *34*, *35*), and ventrally located progenitors are thereby older than more dorsally located siblings at a given time. Consistent with this, down-regulation of *Gli1-3*, *Ptch1*, *Phox2b*, and *Nkx2.9* progressed in an overall V → D fashion but at different kinetics (Fig. 4A). *Gli2* and *Gli3* were most rapidly down-regulated and had, by E10.5, become constrained to the dorsal third of the Nkx2.2^+^ domain, both in WT and Gli1^ON^ mice (Fig. 4, A and B, and fig. S6A). Consistent with the ESC differentiation system, immunoblot analysis revealed that Gli2 and Gli3 proteins were also down-regulated over time in ventral hindbrain tissue (fig. S6C). There was a mutually exclusive relationship between *Tgf*β*2* and *Gli*2*,Gli3* both in control and Gli1^ON^ mice (Fig. 4B). Up-regulation of *Tgf*β*2* in Gli1^ON^ mice was first detected at E10.5 in ventral cells that ceased to express *Gli2* and *Gli3* and which had initiated *Tgf*β*2* expression also in controls but at lower levels (Fig. 4, A and B, and fig. S6B). This shows that a young *Gli2^+^/Gli3*^+^ context is nonpermissive for GliA-mediated *Tgf*β*2* induction, while *Tgf*β*2* responds to GliA in a dose-dependent manner in older *Gli2^−^*/*Gli3^−^* cells. Accordingly, *Gli2^+^/Gli3*^+^ cells must express an inhibitor that acts dominant negative over GliA and that is removed over time, as is the case for GliR (Fig. 2, A and G). In functional support for such a repressor function of GliR, we found that forced expression of GliR was sufficient to suppress GliA-mediated activation of *Tgf*β*2* in epistasis experiments (Fig. 4G). Removal of GliR is thus necessary for activation of *Tgf*β*2*, and the kinetics of its elimination therefore determine the kinetics of *Tgf*β*2* induction. Because of the bifunctional nature of Gli2 and Gli3 proteins, we have not been successful in selectively eliminating GliR in the Nkx2.2^+^ lineage and cannot therefore rule out that early suppression of *Tgf*β*2* may involve repressors in addition to GliR. However, this would not change the overall topology of the timer circuitry, since these hypothetical repressors would then need to be removed either with the same kinetics or faster than GliR to permit activation of *Tgf*β*2* when the GliR concentration declines.

Unexpectedly, in Gli1^ON^ mice, *Phox2b* and *Nkx2.9* expression were augmented in ventral *Gli2^−^/Gli3*^−^ cells but reduced in dorsal *Gli2^+^/Gli3*^+^ cells at E10.5 (Fig. 4B). These transcriptional outputs can only be explained if *Phox2b* and *Nkx2.9* display certain GliR sensitivity, and that the failure to down-regulate *Ptch1* in Gli1^ON^ mice results in reinforced Ptch1-mediated feedback inhibition of the Shh pathway. We observed a notable displacement of Smo out of cilia in Gli1^ON^ conditions over time (Fig. 4, C to E) and increased formation of Gli2R and Gli3R in Gli1^ON^ ESC cultures (Fig. 4F). Thus, increased GliR formation due to Ptch1-mediated feedback begins to suppress *Phox2b* and *Nkx2.9* expression in young Gli2^+^/Gli3^+^ cells in Gli1^ON^ conditions. This cannot occur once cells ceased to express bifunctional Gli proteins, thereby resulting in the anticipated Gli1-mediated up-regulation of *Phox2b* and *Nkx2.9* in older *Gli2^−^/Gli3^−^* cells. Collectively, these data support a model whereby a high GliR sensitivity for *Tgf*β*2* prohibits GliA-mediated activation until GliR has been titrated out, thereby establishing a delayed activation of the Tgfβ node (Fig. 4H). *Phox2b* and other Shh-regulated genes display lower GliR sensitivities (*Tgf*β*2 > Phox2b*, *Nkx2.9 > Ptch1, Gli1*), allowing early activation in the lineage (fig. S7). These data further establish that the decay of GliA followed by down-regulation of Gli genes is functionally important, as it is necessary for evading Ptch1-mediated feedback inhibition, which otherwise begins to interfere with Phox2b expression during the MN window.

### Gli1 is required for *Tgf*β*2* induction and prompt termination of MN production

**Fig. 5:**
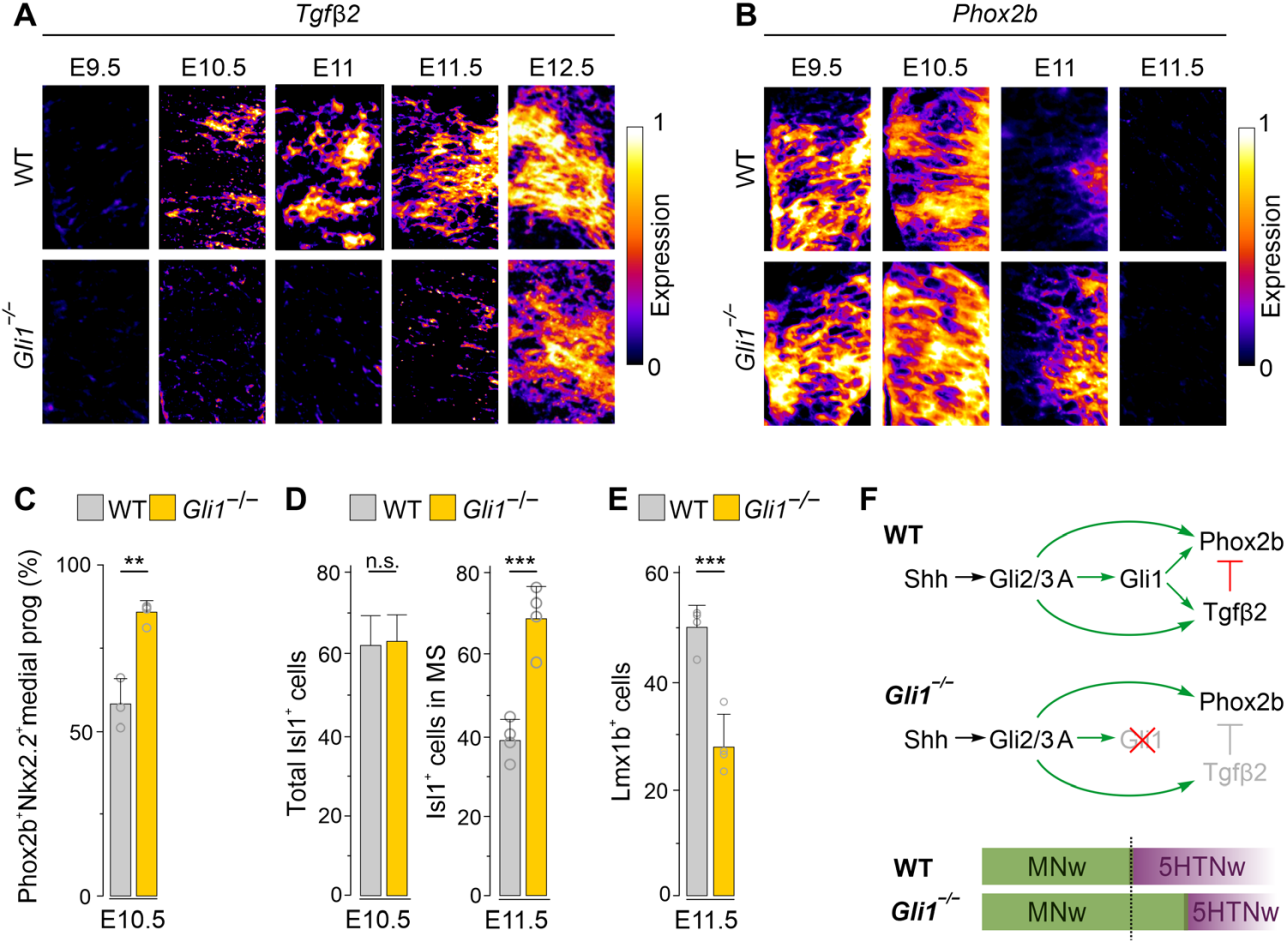
Gli1 is required for timed *Tgf*β*2* induction and correct temporal output. (**A** and **B**) Expression of *Tgf*β*2* and *Phox2b* in Nkx2.2 domain at r2/3 level of WT and *Gli1^−/−^* embryos at indicated stages. (**C** to **E**) Quantification of medially located Nkx2.2^+^ NSCs expressing Phox2b at E10.5, Isl1^+^MNs at E10.5, Isl1^+^MNs in MS at E11.5, and Lmx1b^+^5HTNs at E11.5 in WT and *Gli1^−/−^* embryos. Data from WT *n* = 3 and *Gli1^−/−^ n* = 4 embryos per genotype at E10.5 and from WT and *Gli1*^−/−^ *n* = 4 embryos per genotype at E11.5. Error bars, means ± SD. Asterisks, Student’s *t* test, **P ≤ 0.01 and ***P ≤ 0.001. (**F**) Summary of response properties of *Tgf*β*2* and *Phox2b* to GliA activity.

A Gli inhibitor-titration regulation of *Tgf*β*2* requires that GliA levels remain high enough to activate *Tgf*β*2* once GliR has been titrated away, raising the possibility that the feed-forward activation of *Gli1* by bifunctional Gli proteins functions to boost GliA activity late in the differentiation process. In direct support for this, we found that *Tgf*β*2* failed to be induced on time in *Gli1*^−/−^ mice (Fig. 5A). This was accompanied by prolonged expression of *Phox2b* in progenitors (Fig. 5, B and C, and fig. S8A), a moderate overproduction of Isl1^+^MNs and concurrent reduction of Lmx1b^+^ 5HTNs at E11.5 (Fig. 5, D and E) and *Pet1*^+^ 5HTNs at E12.5 (fig. S8B). *Tgf*β*2* expression had been initiated at low levels by E12.5 in *Gli1* mutants (Fig. 5A), presumably reflecting input by remaining Gli2/3A activity followed by propagation of *Tgf*β*2* expression by positive feedback signaling (Fig. 3B) (*15*). This shows that Gli1 is required for timed activation of *Tgf*β*2* and prompt termination of *Phox2b* and MN production (Fig. 5F), suggesting that Gli2A and Gli3A are the primary activators of *Phox2b*, while timed induction of *Tgf*β*2* also depends on Gli1. In addition, considering that Gli1 binds to DNA with lower affinity than Gli2 (*36*) and *Tgf*β*2* is a direct target of Gli proteins (*37*), this provides a mechanistic rationale for why *Tgf*β*2* is not accessible for Gli1-mediated induction until Gli2R/Gli3R are depleted.

### Robust temporal switch by hysteresis and spatial averaging

**Fig. 6:**
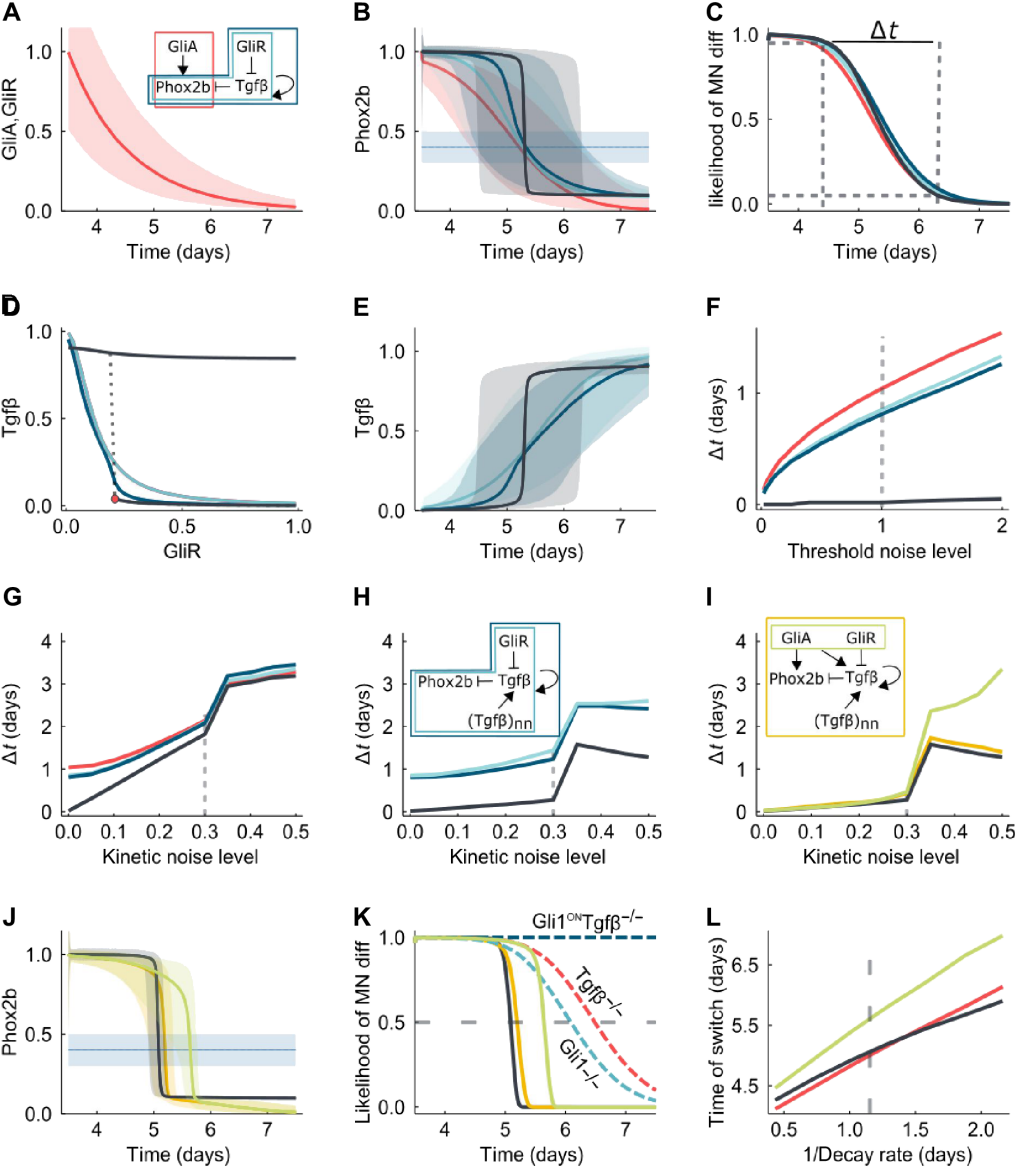
Robust temporal switch by hysteresis and spatial averaging. (**A**) GliA and GliR decay over time. Red line, median values; shaded area, 90% CI for 10^4^ simulations in the presence of kinetic noise. (**B**) Predicted Phox2b concentration for regulatory networks shown in (A) and in case of hysteresis (black). Horizontal line and shadow, noisy threshold for motor-neuron (MN) differentiation. (**C**) Phox2b-dependent likelihood of MN differentiation. (**D**) Bifurcation diagram of *Tgf*β versus GliR showing sensitivities of models without (light blue) and with (dark blue and black lines) *Tgf*β self-activation. (**E**) Predicted *Tgf*β concentration for the different networks. (**F** to **I**) Time interval to switch from MN-to-5HTN as a function of threshold noise (F) and kinetic noise (G) level for networks in (A), and considering additional Tgfβ spatial averaging (H), and a link from GliA on Phox2b and Tgfβ in the hysteretic model (black line) (I), where noise in GliA and GliR is correlated (green) or uncorrelated (yellow). (**J**) Predicted Phox2b concentration for models in (I). (**K**) Likelihood of MN differentiation for models in (I) and indicated mutant mice (dashed lines). (**L**) Time point of switch (differentiation likelihood, 0.5) for different GliA and GliR decay rates. Dashed gray lines in (F to I) and (L) mark the standard value used in simulations.

The timer motif suggested by experimental data relies on the parallel temporal decay of a repressor and an activator, and the delayed activation of a diffusible repressor (Fig. 4H). For all progenitors to undergo a coordinated MN-to-5HTN switch in a short time window, cells have to down-regulate Phox2b synchronously, despite that Gli protein levels vary between cells (Fig. 1, J and K). If we model declining GliA and GliR levels with similar experimental noise levels and a noisy readout threshold (Fig. 6, A and B), then we predict a noisy decline of Phox2b (Fig. 6B) and a transition period from MN-to-5HTN production that lasts for almost 2 days (Fig. 6C), both if we consider a direct regulation of Phox2b by GliA (Fig. 6, B and C, red line) or a relay via GliR and Tgfβ (Fig. 6, B and C, cyan line); we ignore the direct negative impact of GliR on Phox2b as it must be weak, given the strong expression of Phox2b while GliR levels are high. Tgfβ is self-activating, and the introduction of this positive feedback steepens the response (Fig. 6B, blue line), especially when the positive feedback results in a hysteretic switch (Fig. 6B, black line), but does not shorten the transition period Δ*t*, which we define as the time that it takes for the likelihood of MN differentiation to decrease from 0.95 to 0.05 (Fig. 6C). In case of a hysteretic switch, the system is bistable, and as the GliR levels decline, the system reaches a critical point (Fig. 6D, red dot) where it jumps from a low to a high Tgfβ steady state (Fig. 6, D and E, black line). The critical GliR level at which the system returns to the low Tgfβ steady state is at a much higher GliR level, making the switch effectively one way (*38*). Such a hysteretic switch is much more robust to noise in the readout process (Fig. 6F), but this advantage disappears when also considering the impact of kinetic noise (Fig. 6G and fig. S9A), as hysteretic switches are particularly sensitive to kinetic noise (Fig. 6, B and E). Therefore, how can the system achieve a fast, reliable transition? Tgfβ is a short range diffusible protein (*39*) that permits spatial averaging, and this leads to simultaneous fate switching on the population level despite noise (Fig. 6H and fig. S9B). The diffusion range has little impact, and even next-neighbor interactions result in robust synchronized switching at the population level. Note that spatial averaging improves the robustness to noise for all network architectures, but the hysteretic switch achieves a substantially shorter transition period (Fig. 6H and fig. S9B).

*Phox2b* expression is controlled by both GliA and Tgfβ. Addition of the GliA link makes the switch less robust to noise compared to the GliR link alone (via Tgfβ relay), given the lack of spatial averaging. Accordingly, the highest robustness is achieved for a low GliA threshold, as supported by experimental data. Given that generation of Gli2A/Gli3A and Gli2R/Gli3R are coupled (Fig. 2), their levels may be somewhat correlated. However, even perfect correlation (Fig. 6I and fig. S9C, green line) does not improve robustness compared to the uncorrelated case (orange) but does lengthen the time to the switch (Fig. 6, J and K). Thus, a low GliA threshold for activation of Phox2b is important to ensure that the time of Phox2b down-regulation is set by Tgfβ signaling, whose noisiness is reduced by spatial averaging, and reduces the impact of variations of GliA and GliR levels on the duration of the switching interval.

While the timer network is robust to noise, it remains sensitive to structural changes, which allows us to computationally recapitulate the various mutants discussed above (Fig. 6K), thereby confirming the above verbal reasoning that led to the modeled network architecture (Fig. 4H). The combination of a decaying activator and repressor introduces a further save guard as becomes visible when either component is removed (Fig. 6K). Changes in the Gli decay rate (Fig. 6A) alter the time point at which the fate switch occurs (Fig. 6L). In case of an exponential loss of the signal (Fig. 6A), the time point changes proportionally to the inverse of the decay rate (Fig. 6L).

## DISCUSSION

How time is measured by NSCs during temporal patterning of neurons has remained unresolved. In this study, we define a Shh/Gli-driven three-node timer circuitry underlying the sequential specification of MNs and 5HTNs by NSCs. The network is founded on a parallel temporal decay of GliA and GliR established through a progressive down-regulation of *Gli1-3* transcription. Regulatory interactions conform an IFFL circuitry in which GliA not only promotes *Phox2b* expression and MN fate but also accounts for a delayed activation of a suppressive Tgfβ node that triggers a MN-to-5HTN fate switch by repressing *Phox2b*. Data suggest a model in which activation of the Tgfβ node is temporally gated by a Gli inhibitor-titration mechanism, whereby induction of *Tgf*β*2* by Gli1 is prohibited until GliR has been titrated out. We show that GliR is generated also in the absence of Ptch1-mediated feedback inhibition and when the Shh pathway is fully activated (Fig. 2, E and F). Down-regulation of *Gli* genes is consequently necessary for elimination of GliR and initiation of a fate switch by Tgfβ (Figs. 2 and 4H). Changes in the rate of Gli decay alter temporal output by the circuitry and conceptually explain how time is encoded in the lineage. In relation to this, Nkx2.2 has been shown to feedback inhibit *Gli* genes (*35*, *40*), and the MN window is extended in Nkx2.2^−/−^ mice (*12*), suggesting that negative feedback by Nkx2.2 itself impacts on the pace of *Gli* decay and time output. Such feedback modulation of *Gli* genes illustrate how time can be tuned in a lineage-specific manner, which is needed for the generic deployment of the core-timer circuitry in multiple Shh-induced lineages, producing distinct neural progenies over different time frames (*15*, *41*, *42*).

In the adult brain, the transition of dormant NSCs into transient amplifying cells is accompanied by down-regulation of *Gli* genes and is inhibited by GliR (*43*), implying that removal of GliR through adaptation of *Gli* transcription accounts for the transition between these cellular states. Together with our study, this suggests that Gli inhibitor titration and the idea that down-regulation of *Gli* genes facilitates induction through removal of GliR may provide a common mechanism to attain switch-like responses in Shh-regulated differentiation processes.

Biological timers based on accumulation or titration of activators or repressors have previously been reported (*1*, *2*, *4*), and *Gli* decay is formally sufficient to mediate timer function without Tgfβ, raising the question why timing of the MN-to-5HTN fate switch involves a more complex network architecture. Computational modeling suggest that spatial averaging enabled by the diffusible and self-activating properties of Tgfβ, in combination with hysteresis, produces prompt suppression of *Phox2b* and a coordinated switch at the population level (Fig. 6, H and K). Integration of the Tgfβ node thereby counterbalances noise and generates a more precise timer mechanism as compared to a timer based only on *Gli* decay. Spatial averaging further provides potential to reset temporal synchrony at the population level, which should benefit coordination of subsequent fate transitions, as sequentially occurring switches has been shown to be temporally coupled (*15*). These community features are not attainable with temporal networks based exclusively on intrinsic transcriptional regulators. Our data consequently provide a functional basis for the intrinsically programmed activation of extrinsic signals in temporal neural patterning processes, and it seems likely that late-acting extrinsic cues implicated in temporal neurogenesis in the cortex (*18*) function in a manner analogous to Tgfβ.

## MATERIALS AND METHODS

### Mouse models

The following published mouse strains were used in this study: *Nkx6.2::Cre* and *Tgfbr1*^fl/fl^ (*15*), *ROSA26-Gli1^FLAG^* (*31*), *Gli1*^−/−^ (*44*), and *Nkx2.2^−/−^* (*12*). WT and experimental mice were maintained in a C57BL/6 background. Male and female mice were both used depending on availability and were housed in breeding pairs or group-housed with littermates of the same sex after weaning (two to five mice per cage) on a 12-hour light/dark cycle, with food and water provided ad libitum. All experimental procedures were conducted in accordance to the Swedish Animal Agency guidelines for animal experimentation and approved by the regional animal ethics committee of Northern Stockholm.

### Derivation of mouse ESC

Gli1^ON^ ESCs were derived from E3.5 embryos from crossings between *Nkx6.2::Cre* males and *ROSA26-Gli1^FLAG^* females. Briefly, blastocysts were flushed from uterus and washed twice with knockout Dulbecco’s modified Eagle’s medium (KO DMEM) and plated in ESC derivation medium [KO DMEM, 20% knockout serum replacement (KOSR), nonessential amino acids (0.1 mM), GlutaMAX (2 mM), penicillin/streptomycin (100 U/ml), ESGRO® recombinant mouse leukemia inhibitor factor (LIF) supplement (1000 U/ml), and 2-mercaptoethanol (0.1 mM)] containing mitogen-activated protein kinase kinase inhibitor PD0325901 (10 μM) on mitotically inactivated mouse embryonic fibroblasts (MEFs). In 4 to 5 days, blastocysts were attached, inner cell mass outgrowth was dissociated using TrypLE Express (Life Technologies), and cells were plated in ESC derivation medium on mitotically inactivated MEFs. ESCs were passaged two times on MEFs, following propagation in feeder-free conditions in ESC medium [the same as derivation medium but with 12% KOSR and 3% fetal calf serum–embryonic stem (FCS-ES) qualified].

### Differentiation of mESCs into NPCs

Mouse ESCs (mESCs; E14.1) propagated in ESC media [KO DMEM, 12% KOSR, 3% FCS-ES qualified, nonessential amino acids (0.1 mM), GlutaMAX (2 mM), penicillin/streptomycin (100 U/ml), ESGRO (1000 U/ml), and 2-mercaptoethanol (0.1 mM)] under feeder-free condition in a humidified atmosphere containing 5% CO_2_ at 37°C. Stocks of cell lines were free of mycoplasma contamination. For induction of neural differentiation, ESCs were washed twice with phosphate-buffered solution (PBS), dissociated with TrypLE Express, passed through a 40-μm cell strainer, and seeded at a density of 1 × 10^4^ to 2 × 10^4^ cells/cm^2^ on fibronectin-coated surface in N2B27 differentiation medium [50% Neurobasal medium and 50% DMEM/F12, N2 (1:200), B27 (1:100), GlutaMAX (1 mM), penicillin/streptomycin (100 U/ml), bovine serum albumin (25 μg/ml), and 2-mercaptoethanol (0.1 mM)] supplemented with 0.1 μM all-trans retinoic acid (RA) (Sigma-Aldrich) and 0.1 μM Shh-Ag1.3 (SAG). At 3.5DCC, medium was replaced with N2B27 differentiation medium without supplements and changed, subsequently, every second day. To block Shh signaling, 2.5 μM CyC was used as mentioned in the text.

### p-MACS of NPCs

For RNA-seq, qPCR, immunoblotting, and immunoprecipitation experiments neural progenitor cells (NPCs) from 3.5DDC and onward were isolated from differentiating ESCs cultures by Prominin1-based magnetic sorting (p-MACS) according to the manufacturer’s instructions (Miltenyi Biotec). Briefly, cells were dissociated using TrypLE Express, washed twice with ice-cold PBS supplemented with 0.5% bovine serum albumin and 2 mM EDTA (Sigma-Aldrich), and passed through a 40-μm cell strainer. Dissociated cells were incubated with magnetic beads conjugated with anti–prominin-1 antibody. Prominin1^+^ cells were separated on magnetic columns and processed for biochemical analysis. To assess purity of sorted fraction, cells were plated, incubated for 2 hours, and fixed for immunocytochemistry.

### Chick electroporation

Fertilized chick eggs were stored up to 1 week at 16°C, and at the start of experiment, eggs were placed horizontally in a 38°C humidified chamber for ~40 hours until the developing embryo reached a Hamilton-Hamburger stages 11 and 12. Approximately, 5 ml of white was removed to lower the embryo, and an opening in the shell on the top of the egg was made to expose the embryo, which was visualized under a magnifying microscope. A mixture of DNA plasmid(s) and Fasta green (used for coloring) was injected into the central canal of the chick embryo using a pulled capillary needle attached to a mouth aspirator tube. Several drops of PBS were placed on top of the embryos, the electrodes were positioned manually one at each side of the embryos at the hindbrain level, and two 25 ms 6-V electric pulses 1 s apart were applied to the embryo using an Electro square Porator ECM 830. The eggs were sealed and incubated at 38°C for 40 hours before embryo collection and processing for immunofluorescence or in situ hybridization.

### Immunohistochemistry

Embryonic stage of collected mouse embryos was determined by somite number (E9.5 to E11.5 embryos) and by limb anatomy (E10 to E12.5 embryos). Mouse embryos from E9.5 to E11.5 5 and chick embryos collected 40 hours after electroporation were fixed in 4% paraformaldehyde in 0.1 M PBS for 2 hour on ice with shaking, and E12.5 mouse embryos were fixed overnight at 4°C. Fixed tissue was washed with PBS, cryoprotected by equilibration in 30% sucrose in PBS, embedded in O.C.T.^TM^, frozen on dry ice, and cryostat-sectioned in the transverse plane at 12 μm. Tissue sections were collected at hindbrain level, and rhombomeric level was determined by anatomical landmarks (i.e., otic vesicle) and Phox2b/Isl1 immunohistochemistry to visualize MNs. Immuhistochemistry was carried out in water-humidified chamber. The tissue on slides was incubated with blocking solution (3% FCS/0.1% Triton X-100 in PBS) at room temperature (RT) for 1 hour followed by incubation with primary antibodies overnight at 4°C and fluorophore-conjugated secondary antibodies for 1 hour at RT. Both primary and fluorophore-conjugated secondary antibodies were diluted in blocking solution. Sections were mounted using Vectashield and coverslipped for imaging.

For ESC differentiated material, cells were fixed for 12 mins at RT in 4% paraformaldehyde in PBS, rinsed three times in PBST (PBS with 0.1% Triton X-100), and blocked for 1 hour at RT with blocking solution (3% FCS/0.1% Triton X-100 in PBS). Cells were then incubated with primary antibodies overnight at 4°C followed by incubation with fluorophore-conjugated secondary antibodies for 1 hour at RT. Primary antibodies used are as follows: mouse anti-Nkx2.2, Isl1 Developmental Studies Hybridoma Bank (DSHB); mouse anti-Smo, Gata3 (Santa Cruz Biotechnology); mouse anti-Gli1 (Cell Signaling Technology); rabbit anti-Arl13b (ProteinTech Group); mouse anti-Arl13b (NeuroMab); goat anti-Gli2, Gli3, and Sox1 (R&D Systems); guinea-pig anti-Gli2 (gift from J. Eggenschwiler, University of Georgia); guinea-pig anti-Phox2b, Lmx1b, and Nkx2.9 (homemade); mouse anti-actin (Seven Hills Bioreagents); and rabbit anti-Arx (gift from K. Miyabayashi). Sections were mounted using Mowiol/DABCO mounting solution. Images were captured using Zeiss LSM880 confocal microscope. In Fig. 1H, Arl13 immunostaining presented with Nkx2.2 and with Gli2 correspond to a triple immunocytochemistry with antibodies against Arl13b and Nkx2.2 with Gli2. Similarly, in fig. S1 (C and D), images presented correspond to a triple immunocytochemistry with antibodies against Arl13b and Nkx2.2 with Gli3 (fig. S1C) or with Gli1 (fig. S1D) and where Arl13 immunostaining is presented with Nkx2.2 and with Gli3 or Gli1.

### In situ hybridization

Mounted tissue sections were postfixed in 4% paraformaldehyde (PFA) in phosphate buffer for 10 min, washed three times for 5 min with PBS, incubated with proteinase K solution [1 μg/ml proteinase K in 50 mM tris-Cl (pH7.5) and 5 mM EDTA] for 5 min and PFA-fixed and washed with PBS as before. Slides were subsequently treated with an acetylating solution [2 mM HCl water solution containing triethanolamide (14 μl/ml) and acetic anhydride (2.5 μl/ml)] for 10 min, washed three times with PBS, and incubated with hybridization solution [50% formamide, 5× saline-sodium citrate (SSC), 5× denharts, yeast RNA (250 μg/ml), herring sperm DNA (500 μg/ml), and blocking reagent from Roche (20 mg/ml)] for 1 hour in a chamber humidified with a 50% formamide/5× SSC solution. All steps were performed at RT. Slides were subsequently incubated overnight at 70°C with labeled probe diluted in hybridization solution, followed by washes with a 0.2× SSC solution, first at 70°C for 1 hour and then by another for 10 min at RT. Slides were incubated in B1 solution [0.1 M tris-Cl (pH7.5), 0.15 M NaCl, and 10% heat inactivated fetal bovine serum (FBS)] for 1 hour at RT, followed by an overnight incubation with anti–digoxigenin (DIG)–alkaline phosphatase Fab fragments antibody diluted in B1 solution at 4°C in a water humidified chamber. Excess antibody was washed away with three washes at RT with B1 solution and equilibrated in B3 solution [0.1 M tris-Cl (pH 9.5), 0.1 M NaCl, and 50 mM MgCl_2_] before incubation of tissue with developing solution [10% polyvinyl alcohol, 100 mM tris-Cl (pH 9.5), 100 mM NaCl, 5 mM MgCl_2_, and levamisol (0.24 mg/ml)] containing nitro blue tetrazolium/bromochloroindolyl phosphate (Roche) at RT. The developing reaction was stopped by placing the slides in water and after several washes, and slides were mounted using Aquatex mounting medium (Merck). Labeled probes were produced from in vitro transcription of linearized plasmid containing a sequence specific for the desired gene using a DIG RNA labeling kit (Roche) according to the manufacturer’s protocol. Probes were purified using Microspin G-50 columns (GE Healthcare) and stored at −20°C.

### EdU Labeling

EdU (Life Technologies) was injected intraperitoneally in pregnant female mice at 0.04 mg/g of body weight and detected using the Click-iT EdU Alexa Fluor 555 Imaging Kit (Life Technologies) according to the manufacturer’s protocol.

### Isolation of mouse neural tissue for qPCR and Western blot

Neural tube tissue was isolated as described before (*45*). In brief, mouse embryonic tissue was dissected in L-15 medium and staged based on somite number and limb morphology, and hindbrain region was roughly dissected out with the help of tungsten needles. To completely remove the mesenchymal tissue surrounding the neural tissue, the dissected hindbrain piece was treated sequentially with dispase solution [dispase (1 mg/ml) in L-15 medium] for 5 min, 10% FBS solution (in L-15 medium) for 5 min and washed twice in L-15 medium. The remaining mesenchymal tissue was then removed with the help of tungsten needles. For qPCR analysis, the ventral region of rhombomeres 2 and 3 of the hindbrain was isolated, while for Western blot analysis, the ventral and intermediate domains of the hindbrain were collected. At the stages analyzed, the rhombomeric units of the hindbrain can be easily defined by grooves in the neural tissue. RNA for each individual piece was isolated using the RNeasy Micro Kit with a deoxyribonuclease digestion step (Qiagen) according to the manufacturer’s protocol. Whole-cell protein extracts and Western blot were performed as described in the “Immunoblotting and immunoprecipitation” section.

### Immunoblotting and immunoprecipitation

For whole-cell extracts, p-MACS isolated NPCs were lysed in radioimmunoprecipitation assay buffer (Sigma-Aldrich) complemented with protease and phosphatase inhibitor cocktail (Thermo Fisher Scientific) and incubated on ice with shaking for 30 min. Lysate was cleared by centrifugation (20,000*g* for 20 min at 4°C) and protein concentration determined by bicinchoninic acid assay. Protein lysate was resuspended in NuPAGE^TM^ LSD sample buffer (Thermo Fisher Scientific) with 2.5% 2-mercaptoethanol and denaturated at 95°C for 5 min. Fifteen to 30 μg of protein were loaded per lane of a 10% SDS polyacrylamide gel (Bio-Rad) and transferred onto nitrocellulose membranes (Bio-Rad) using a Trans-Blot Turbo System (Bio-Rad). Membranes were incubated 1 hour in blocking solution [tris-buffered saline with 0.1% Tween 20 (TBST) and 5% nonfat dry milk], followed by overnight incubation at 4°C with primary antibodies. After three washes with TBST, membranes were incubated with horseradish peroxidase (HRP)–conjugated secondary antibodies for 1 hour at RT. Detection of HRP was performed by chemiluminescent substrate SuperSignal West Dura substrate, and signal was detected on a ChemiDoc Imaging System (Bio-Rad).

For immunoprecipitation assays, 300 μg of protein lysate was diluted with Pierce IP lysis buffer (Thermo Fisher Scientific) up to 1 ml and incubated overnight at 4°C with 2 μg of primary antibody. Protein-antibody complex was isolated with protein A/G magnetic beads according to the manufacturer’s instructions (Thermo Fisher Scientific). Precipitated material was used for immunoblotting as described above. Primary antibodies used are as follows: mouse anti-Gli1 (Cell Signaling Technology), goat anti-Gli2, Gli3 (R&D Systems), mouse anti–glyceraldehyde-3-phosphate dehydrogenase (GAPDH) (Thermo Fisher Scientific), and mouse anti-actin (Seven Hills Bioreagents).

### Image analysis

For quantification of protein level from immunofluorescence, confocal images were analyzed in ImageJ 1.48v. Nuclear area was defined by 4′,6-diamidino-2-phenylindole, Nkx2.2, or Sox1 immunostaining, and integrated density of fluorescent signal in individual nuclei was determined.

Images from in situ hybridization were acquired in a Zeiss Axio Imager.M2 microscope coupled to an Axio camera 503 mono and processed in Photoshop CS6. Images in Fig. 5 (A and B) were processed in ImageJ 1.48v using LUT-Fire function.

To determine relative gene expression levels along the Nkx2.2 domain (Fig. 4), images were analyzed in ImageJ 1.48v using “Plot Profile” function. A region of tissue that did not express the gene was used to determine background levels.

Western blots were quantified in ImageJ 1.48v using Gel Analysis Tool.

### RNA extraction, cDNA preparation, and qPCR

Total RNA was isolated from cells using an RNeasy Mini kit (Qiagen) or Quick-RNA MiniPrep Plus kit (Zymo Research). RNA (500 ng) was used for complementary DNA (cDNA) preparation using a Maxima First Strand cDNA synthesis kit (Thermo Fisher Scientific). Quantitative real-time PCR was performed in a 7500 Fast Real Time PCR system thermal cycler with Fast SYBR Green PCR Master Mix (Applied Biosystems). Analysis of gene expression was performed using the 2-ΔΔ*Ct* method, and relative gene expression was normalized to *Gapdh* transcript levels using primer for mouse: *Gli1* (forward, 5′-gtcggaagtc ctattcacgc-3′; reverse, 5′-cagtctgctctcttccctgc-3′), *Gli2* (forward, 5′-agctccacacacccgcaaca-3′; reverse, 5′-tgcagctggctcagcatcgt-3′), *Gli3* (forward, 5′-caaccacagcccttgctttgc-3′; reverse, 5′-ggcccacccg agctatagttg-3′), *Gli1* (5′ untranslated region) (forward, 5′-cctttcttgaggttgggatgaag-3′; reverse, 5′-gcgtctcagggaaggatga-3′), *Ptch1* (forward, 5′-actgtccagctaccccaatg-3′ ; reverse, 5′-catcatgccaaagagctcaa-3′), *Ptch2* (forward, 5′-cctagaacagctctgggtagaagt-3′ ; reverse, 5′-cccagcttctccttggtgta-3′), *Nkx2.9* (forward, 5′-gtgcgttccacagactgct-3′; reverse, 5′-gagtctgcagggcttgtctc-3′), *Phox2b* (forward, 5′-tgagacgcactaccctgaca-3′; reverse, 5′-cggttctggaaccacacct-3′), *Gapdh* (forward, 5′-gtggtgaagcaggcatctga-3′; reverse, 5′-gccatgtaggccatgaggtc-3′), and *Tgf*β*2* [predesigned from Integrated DNA Technologies (IDT) toward exon 4/5].

### RNA-seq, gene expression quantification, and differential expression analysis

RNA concentration and integrity were determined on an Agilent RNA 6000 Pico chip using Agilent 2100 BioAnalyzer (Agilent Technologies). RNA-seq of condition-specific samples was performed by the National Genomics Infrastructure at the Science for Life Laboratory in Stockholm on Illumina HiSeq2500 in RapidHighOutput mode with single-end setup (1× 50–base pair read length). Reads were mapped to the mouse genome assembly build GRCm38 using TopHat (v2.0.4). Next, the number of reads mapped to each gene was calculated using htseq-count (v0.6.1). The gene level abundances were estimated as fragments per kilobase of transcript per million mapped reads (FPKMs) using Cufflinks (v2.1.1). Plots and histograms were made using FPKM values. Furthermore, we processed the read count data with RNA-seq–specific functions of R package limma. For adjusting the mean-variance dependence, we used the function voom (*46*), which rendered the expression data into normally distributed log_2_ counts per million values, accompanied with observation-level gene-specific adjusting weights. The differential expression was estimated with functions lmFit, eBayes, and topTable. The variance estimates were obtained by treating all samples as replicates (design = NULL) and obtaining library sizes from counts (lib.size = NULL) without further normalization (normalize.method = “none”). The output FC values of differential expression were accompanied with *P* values. The latter were adjusted for multiple testing by calculating Benjamini-Hochberg’s false discovery rate (*47*).

### Identification of genes changing over time

To determine genes down-regulated in NSCs between 3.5DDC and 5.5DDC, FPKM values at each time point, *P* values, and FC values calculated as described above were used, and the following criteria were applied: FPKM (3.5DDC) ≥ 2, *P* ≤ 0.05, and down-regulated (log_2_FC ≥ 0.22). In addition, only protein-coding genes were considered.

### Determination of biphasic genes

To identify genes that exhibited a biphasic expression profile during differentiation (from 0 to 6.5 DDC) and that were repressed by CyC treatment, we determined genes, whose expression peaked at 2 or 3.5 DDC and were repressed in 3.5DDC progenitors differentiated in CyC conditions. An initial criterion was applied as a cutoff of gene expression and Cyc sensitivity [FPKM at 2DDC or 3.5DDC ≥ 2, *P* value at 3.5DDC between SAG- and CyC-treated cultures ≤ 0.05, and log_2_FC at 3.5DDC between SAG- and CyC-treated cultures ≥ 0.5 (down-regulated)]. Next, genes whose expression peaked at 2 DDC or 3.5 DCC were determined using the following criteria. Peak at 2DDC: differentially expressed between 2DDC and 6.5DDC and 0DDC (*P* ≤ 0.05), up-regulated between 0DDC and 2DDC [log_2_FC(2-0DDC) ≥ 0.5, log_2_FC(2-1DDC) ≥ 0], and down-regulated between 2DDC and 6.5DDC [log_2_FC(2–6.5DDC) ≥ 0.5, log_2_FC(2-5.5DDC) ≥ 0.5, log_2_FC(2–4.5DDC) ≥ 0]. Peak at 3.5DDD: differentially expressed between 3.5DDC and 6.5DDC and 0DDC (*P* ≤ 0.05), up-regulated between 0DDC and 3.5DDC [log_2_FC(3.5–0DDC) ≥0.5, log_2_FC(3.5–1DDC) ≥ 0, log_2_FC(2–1DDC) ≥ 0], and down-regulated between 3.5DDC and 6.5DDC [log_2_FC(3.5–6.5DDC) ≥ 0.5, log_2_FC(3.5–5.5DDC) ≥ 0.5]. In addition, only protein-coding genes were considered.

### Theoretical framework

Our theoretical framework considers the dynamics of GliA (*A*), GliR (*R*), Tgfβ (*T*), and Phox2b (*P*). We approximate the measured dynamics of GliA and GliR (Fig. 1N) by an exponential decay from initial values *A*(0) = *R*(0) =1

$$\frac{\mathrm{dA}}{\mathrm{dt}}=-\lambda_{A}\;A$$ (1)

$$\frac{\mathrm{dR}}{\mathrm{dt}}=-\lambda_{R}\;R$$ (2)

The equations for Tgfβ and Phox2b differ for the different regulatory networks. A generalized form is given by

$$\frac{\mathrm{dT}}{\mathrm{dt}}=v_{T}[(1-\omega_{\mathrm{TT}})H^{w+}(A,h_{\mathrm{AT}},\omega_{\mathrm{AT}})H^{w-}(R,h_{\mathrm{RT}},\omega_{\mathrm{RT}})+\omega_{\mathrm{TT}}H^{w+}(T,h_{\mathrm{TT}},\omega_{\mathrm{TT}})-T]$$ (3)

$$\frac{\mathrm{dP}}{\mathrm{dt}}=v_{P}[H^{w+}(A,h_{\mathrm{AP}},\omega_{\mathrm{AP}})H^{w-}(T,h_{\mathrm{TP}},\omega_{\mathrm{TP}})-P]$$ (4)

where the functions *H*^*w*+^(*x*, *h*, ω) = (1 − ω) + ω*H*^+^(*x*, *h*) and *H*^*w*−^(*x*, *h*, ω) = (1 − ω) + ω*H*^−^(*x*, *h*) represent weighted positive and negative Hill functions, respectively. For all Hill functions, we use a Hill factor equal to 2, i.e., $H^{+}(x,h)=\frac{x^{2}}{h^{2}+x^{2}}$ and $H^{-}(x,h)=\frac{h^{2}}{h^{2}+x^{2}}$. The different links in the regulatory networks are represented via ω (Table 1). Thus, in the case of ω = 0, *H*^*w*+^ = 1, and *H*^*w*−^ = 1, and the link represented by this function is not being considered. In the case of ω = 1, *H*^*w*+^ = *H*^+^, and *H*^*w*−^ = *H*^−^ represent a positive and negative Hill function, respectively. We use *v_T_* as production and decay rates, so that the maximal concentrations Tgfβ and Phox2b is one and their values are restricted to the interval [0, 1].

**Table 1:** Parameter values used in the simulations.

**Model**	**ω*_AT_***	**ω*_RT_***	**ω*_TT_***	**ω*_AP_***	**ω*_TP_***	***h_AT_***	***h_RT_***	***h_TT_***	***h_AP_***	***h_TP_***
GliA -> Phox2b	0.0	0.0	0.0	1.0	0.0	–	-	–	0.25	–
GliR-| Tgfβ -| Phox2b	0.0	1.0	0.0	0.0	1.0	–	0.15	–	–	0.3
GliR-| Tgfβ -| Phox2b (with self- activation)	0.0	1.0	0.4	0.0	1.0	–	0.1	0.3	–	0.3
GliR-| Tgfβ -| Phox2b (with hysteresis)	0.0	1.0	0.94	0.0	1.0	–	0.2	0.3	–	0.3
Full network (WT)	1.0	1.0	0.94	1.0	1.0	0.08	0.2	0.3	0.08	0.3

To represent the effects of molecular noise, we added scalar white noise to the equations, i.e., a noise term that is proportional to the values of the variables. Therefore, the ordinary differential equations of the form

dx=f(x,y,z)dt

become stochastic differential equations of the form

dx=f(x,y,z)dt+ε x dW

where *W* denotes a Wiener process and ε denotes the kinetic noise level.

In Fig. 6 (I to K), we have considered both correlated and uncorrelated kinetic noise in GliA and GliR. In the correlated case (green lines), we used the same values for GliA and GliR, while in the uncorrelated case (yellow lines), the values are obtained by independently solving Eqs. 1 and 2, with a noise term.

To evaluate the impact of noise in the Phox2b concentration on the likelihood of a cell to differentiate into either a MN or 5HTN, we defined the likelihood of MN differentiation as the cumulative distribution function of a gamma distribution with different levels of variance. More specifically, we defined the shape (*k*) and the scale (θ) parameters of the gamma distribution as *k* = 10/η and θ = 0.04 η, where η represents the threshold noise level. The average of this gamma distribution is given by *k*θ = 0.4 for all η, while the variance is proportional to η and given by 1.6 10^−2^ η.

The effect of Tgfβ diffusion (Fig. 6, H to K) was introduced by replacing the variable *T* in the term *H*^*w*+^(*T*, *h_TT_*, ω*_TT_*) in Eq. 3 and the term *H*^*w*−^(*T*, *h_TP_*, ω*_TP_*) in Eq. 4 with 0.5*T* + 0.5〈*T*〉*_nn_*, where 〈*T*〉*_nn_* represents the average values of Tgfβ in the nearest neighboring cells. Averaging over a wider range of neighbors yields similar results. In our simulations, we have considered an array of 100 × 100 cells with periodic boundary conditions.

#### Mutants

In the table, the full network represents the WT case. For the *Tgfbr1*^−/−^ mutant, we removed the Tgfβ modulation on Phox2b by setting ω*_TP_* = 0.0. On the basis of data in Fig. 2, we used a 30% lower starting value for GliA in the Gli1^−/−^ mutant compared to WT. Last, in the Gli^ON^Tgfbr1^−/−^ mutant, the GliA concentration remains always well above the threshold for Phox2b production. Given that Tgfβ is absent, the Phox2b concentration always remains above the threshold for MN formation.

#### Parameter values

In the simulations, we have used λ*_A_* = λ*_R_* = 0.86, *v_T_* = 50, and *v_P_* = 90. The other parameter values vary according to the model and are presented in table below.

### Statistical analysis

Quantitative data are reported as means ± SD or as means ± SEM or as box plots (with whiskers entending to 5th and 95th percentile) as specified in figure legends. The Student’s *t* test or pairwise Wilcoxon test was used for evaluation of significance as specified in figure legends. The sample number (*n*) indicated in figure legends refers to the number of biological replicates. n.s. = not significant (*P* > 0.05), *P ≤ 0.05, **P ≤ 0.01, and ***P ≤ 0.001.

## Supplementary Material

aba8196_SM.pdf

## SUPPLEMENTARY MATERIALS

Supplementary material for this article is available at http://advances.sciencemag.org/cgi/content/full/6/38/eaba8196/DC1

## Appendix

View/request a protocol for this paper from *Bio-protocol*.

## References

[1] Averbukh I., Lai S.-L., Doe C. Q., Barkai N., A repressor-decay timer for robust temporal patterning in embryonic *Drosophila* neuroblast lineages. eLife 7, e38631 (2018). 30526852 10.7554/eLife.38631 PMC6303102

[2] Collart C., Allen G. E., Bradshaw C. R., Smith J. C., Zegerman P., Titration of four replication factors is essential for the *Xenopus laevis* midblastula transition. Science 341, 893–896 (2013). 23907533 10.1126/science.1241530 PMC3898016

[3] Ebisuya M., Briscoe J., What does time mean in development? Development 145, dev164368 (2018). 29945985 10.1242/dev.164368 PMC6031406

[4] Dugas J. C., Ibrahim A., Barres B. A., A crucial role for p57(Kip2) in the intracellular timer that controls oligodendrocyte differentiation. J. Neurosci. 27, 6185–6196 (2007). 17553990 10.1523/JNEUROSCI.0628-07.2007 PMC6672145

[5] Lodato S., Arlotta P., Generating neuronal diversity in the mammalian cerebral cortex. Annu. Rev. Cell Dev. Biol. 31, 699–720 (2015). 26359774 10.1146/annurev-cellbio-100814-125353 PMC4778709

[6] Telley L., Agirman G., Prados J., Amberg N., Fièvre S., Oberst P., Bartolini G., Vitali I., Cadilhac C., Hippenmeyer S., Nguyen L., Dayer A., Jabaudon D., Temporal patterning of apical progenitors and their daughter neurons in the developing neocortex. Science 364, eaav2522 (2019). 31073041 10.1126/science.aav2522

[7] Rossi A. M., Fernandes V. M., Desplan C., Timing temporal transitions during brain development. Curr. Opin. Neurobiol. 42, 84–92 (2017). 27984764 10.1016/j.conb.2016.11.010 PMC5316342

[8] Li X., Chen Z., Desplan C., Temporal patterning of neural progenitors in Drosophila. Curr. Top. Dev. Biol. 105, 69–96 (2013). 23962839 10.1016/B978-0-12-396968-2.00003-8 PMC3927947

[9] Angevine J. B. Jr., Sidman R. L., Autoradiographic study of cell migration during histogenesis of cerebral cortex in the mouse. Nature 192, 766–768 (1961). 10.1038/192766b0 17533671

[10] Rakic P., Neurons in rhesus monkey visual cortex: Systematic relation between time of origin and eventual disposition. Science 183, 425–427 (1974). 4203022 10.1126/science.183.4123.425

[11] Syed M. H., Mark B., Doe C. Q., Playing well with others: Extrinsic cues regulate neural progenitor temporal identity to generate neuronal diversity. Trends Genet. 33, 933–942 (2017). 28899597 10.1016/j.tig.2017.08.005 PMC5701851

[12] Pattyn A., Vallstedt A., Dias J. M., Samad O. A., Krumlauf R., Rijli F. M., Brunet J. F., Ericson J., Coordinated temporal and spatial control of motor neuron and serotonergic neuron generation from a common pool of CNS progenitors. Genes Dev. 17, 729–737 (2003). 12651891 10.1101/gad.255803 PMC196019

[13] Kohwi M., Doe C. Q., Temporal fate specification and neural progenitor competence during development. Nat. Rev. Neurosci. 14, 823–838 (2013). 24400340 10.1038/nrn3618 PMC3951856

[14] Vallstedt A., Klos J. M., Ericson J., Multiple dorsoventral origins of oligodendrocyte generation in the spinal cord and hindbrain. Neuron 45, 55–67 (2005). 15629702 10.1016/j.neuron.2004.12.026

[15] Dias J. M., Alekseenko Z., Applequist J. M., Ericson J., Tgfβ signaling regulates temporal neurogenesis and potency of neural stem cells in the CNS. Neuron 84, 927–939 (2014). 25467979 10.1016/j.neuron.2014.10.033

[16] D’Autréaux F., Coppola E., Hirsch M.-R., Birchmeier C., Brunet J.-F., Homeoprotein Phox2b commands a somatic-to-visceral switch in cranial sensory pathways. Proc. Natl. Acad. Sci. U.S.A. 108, 20018–20023 (2011). 22128334 10.1073/pnas.1110416108 PMC3250195

[17] Shen Q., Wang Y., Dimos J. T., Fasano C. A., Phoenix T. N., Lemischka I. R., Ivanova N. B., Stifani S., Morrisey E. E., Temple S., The timing of cortical neurogenesis is encoded within lineages of individual progenitor cells. Nat. Neurosci. 9, 743–751 (2006). 16680166 10.1038/nn1694

[18] McConnell S. K., Kaznowski C. E., Cell cycle dependence of laminar determination in developing neocortex. Science 254, 282–285 (1991). 1925583 10.1126/science.254.5029.282

[19] Jacob J., Ferri A. L., Milton C., Prin F., Pla P., Lin W., Gavalas A., Ang S.-L., Briscoe J., Transcriptional repression coordinates the temporal switch from motor to serotonergic neurogenesis. Nat. Neurosci. 10, 1433–1439 (2007). 17922007 10.1038/nn1985

[20] Pattyn A., Simplicio N., van Doorninck J. H., Goridis C., Guillemot F., Brunet J.-F., *Ascl1/Mash1* is required for the development of central serotonergic neurons. Nat. Neurosci. 7, 589–595 (2004). 15133515 10.1038/nn1247

[21] Pattyn A., Hirsch M., Goridis C., Brunet J. F., Control of hindbrain motor neuron differentiation by the homeobox gene Phox2b. Development 127, 1349–1358 (2000). 10704382 10.1242/dev.127.7.1349

[22] Okamoto M., Miyata T., Konno D., Ueda H. R., Kasukawa T., Hashimoto M., Matsuzaki F., Kawaguchi A., Cell-cycle-independent transitions in temporal identity of mammalian neural progenitor cells. Nat. Commun. 7, 11349 (2016). 27094546 10.1038/ncomms11349 PMC4842982

[23] Briscoe J., Thérond P. P., The mechanisms of Hedgehog signalling and its roles in development and disease. Nat. Rev. Mol. Cell Biol. 14, 416–429 (2013). 23719536 10.1038/nrm3598

[24] Matise M. P., Wang H., Sonic hedgehog signaling in the developing CNS: Where it has been and where it is going. Curr. Top. Dev. Biol. 97, 75–117 (2011). 22074603 10.1016/B978-0-12-385975-4.00010-3

[25] Peterson K. A., Nishi Y., Ma W., Vedenko A., Shokri L., Zhang X., McFarlane M., Baizabal J.-M., Junker J. P., van Oudenaarden A., Mikkelsen T., Bernstein B. E., Bailey T. L., Bulyk M. L., Wong W. H., McMahon A. P., Neural-specific Sox2 input and differential Gli-binding affinity provide context and positional information in Shh-directed neural patterning. Genes Dev. 26, 2802–2816 (2012). 23249739 10.1101/gad.207142.112 PMC3533082

[26] Vokes S. A., Ji H., Wong W. H., McMahon A. P., A genome-scale analysis of the cis-regulatory circuitry underlying sonic hedgehog-mediated patterning of the mammalian limb. Genes Dev. 22, 2651–2663 (2008). 18832070 10.1101/gad.1693008 PMC2559910

[27] Oosterveen T., Kurdija S., Alekseenko Z., Uhde C. W., Bergsland M., Sandberg M., Andersson E., Dias J. M., Muhr J., Ericson J., Mechanistic differences in the transcriptional interpretation of local and long-range Shh morphogen signaling. Dev. Cell 23, 1006–1019 (2012). 23153497 10.1016/j.devcel.2012.09.015

[28] Yu K., McGlynn S., Matise M. P., Floor plate-derived sonic hedgehog regulates glial and ependymal cell fates in the developing spinal cord. Development 140, 1594–1604 (2013). 23482494 10.1242/dev.090845 PMC3596997

[29] Humke E. W., Dorn K. V., Milenkovic L., Scott M. P., Rohatgi R., The output of Hedgehog signaling is controlled by the dynamic association between Suppressor of Fused and the Gli proteins. Genes Dev. 24, 670–682 (2010). 20360384 10.1101/gad.1902910 PMC2849124

[30] Alfaro A. C., Roberts B., Kwong L., Bijlsma M. F., Roelink H., Ptch2 mediates the Shh response in *Ptch^1−/−^* cells. Development 141, 3331–3339 (2014). 25085974 10.1242/dev.110056 PMC4199129

[31] Vokes S. A., Ji H., McCuine S., Tenzen T., Giles S., Zhong S., Longabaugh W. J. R., Davidson E. H., Wong W. H., McMahon A. P., Genomic characterization of Gli-activator targets in sonic hedgehog-mediated neural patterning. Development 134, 1977–1989 (2007). 17442700 10.1242/dev.001966

[32] Buchler N. E., Louis M., Molecular titration and ultrasensitivity in regulatory networks. J. Mol. Biol. 384, 1106–1119 (2008). 18938177 10.1016/j.jmb.2008.09.079

[33] Zhang Q., Bhattacharya S., Andersen M. E., Ultrasensitive response motifs: Basic amplifiers in molecular signalling networks. Open Biol. 3, 130031 (2013). 23615029 10.1098/rsob.130031 PMC3718334

[34] Dessaud E., Yang L. L., Hill K., Cox B., Ulloa F., Ribeiro A., Mynett A., Novitch B. G., Briscoe J., Interpretation of the sonic hedgehog morphogen gradient by a temporal adaptation mechanism. Nature 450, 717–720 (2007). 18046410 10.1038/nature06347

[35] Lek M., Dias J. M., Marklund U., Uhde C. W., Kurdija S., Lei Q., Sussel L., Rubenstein J. L., Matise M. P., Arnold H.-H., Jessell T. M., Ericson J., A homeodomain feedback circuit underlies step-function interpretation of a Shh morphogen gradient during ventral neural patterning. Development 137, 4051–4060 (2010). 21062862 10.1242/dev.054288

[36] Taylor R., Long J., Yoon J. W., Childs R., Sylvestersen K. B., Nielsen M. L., Leong K.-F., Iannaccone S., Walterhouse D. O., Robbins D. J., Iannaccone P., Regulation of GLI1 by cis DNA elements and epigenetic marks. DNA Repair 79, 10–21 (2019). 31085420 10.1016/j.dnarep.2019.04.011 PMC6570425

[37] Ali S. A., Niu B., Cheah K. S. E., Alman B., Unique and overlapping GLI1 and GLI2 transcriptional targets in neoplastic chondrocytes. PLOS ONE 14, e0211333 (2019). 30695055 10.1371/journal.pone.0211333 PMC6350985

[38] Tyson J. J., Chen K. C., Novak B., Sniffers, buzzers, toggles and blinkers: Dynamics of regulatory and signaling pathways in the cell. Curr. Opin. Cell Biol. 15, 221–231 (2003). 12648679 10.1016/s0955-0674(03)00017-6

[39] Massagué J., TGFβ signalling in context. Nat. Rev. Mol. Cell Biol. 13, 616–630 (2012). 22992590 10.1038/nrm3434 PMC4027049

[40] Nishi Y., Zhang X., Jeong J., Peterson K. A., Vedenko A., Bulyk M. L., Hide W. A., McMahon A. P., A direct fate exclusion mechanism by Sonic hedgehog-regulated transcriptional repressors. Development 142, 3286–3293 (2015). 26293298 10.1242/dev.124636 PMC4631756

[41] Deng Q., Andersson E., Hedlund E., Alekseenko Z., Coppola E., Panman L., Millonig J. H., Brunet J.-F., Ericson J., Perlmann T., Specific and integrated roles of Lmx1a, Lmx1b and Phox2a in ventral midbrain development. Development 138, 3399–3408 (2011). 21752929 10.1242/dev.065482

[42] Miyoshi G., Butt S. J. B., Takebayashi H., Fishell G., Physiologically distinct temporal cohorts of cortical interneurons arise from telencephalic *Olig2*-expressing precursors. J. Neurosci. 27, 7786–7798 (2007). 17634372 10.1523/JNEUROSCI.1807-07.2007 PMC6672881

[43] Petrova R., Garcia A. D. R., Joyner A. L., Titration of GLI3 repressor activity by sonic hedgehog signaling is critical for maintaining multiple adult neural stem cell and astrocyte functions. J. Neurosci. 33, 17490–17505 (2013). 24174682 10.1523/JNEUROSCI.2042-13.2013 PMC3812512

[44] Park H. L., Bai C., Platt K. A., Matise M. P., Beeghly A., Hui C. C., Nakashima M., Joyner A. L., Mouse Gli1 mutants are viable but have defects in SHH signaling in combination with a Gli2 mutation. Development 127, 1593–1605 (2000). 10725236 10.1242/dev.127.8.1593

[45] Alekseenko Z., Andersson E., Dias J., Chick neural tube explant culture. Bio-protocol 5, e10608 (2015).

[46] Law C. W., Chen Y., Shi W., Smyth G. K., voom: Precision weights unlock linear model analysis tools for RNA-seq read counts. Genome Biol. 15, R29 (2014). 24485249 10.1186/gb-2014-15-2-r29 PMC4053721

[47] Benjamini Y., Hochberg Y., Controlling the false discovery rate: A practical and powerful approach to multiple testing. J. R. Stat. Soc. Ser. B. 57, 289–300 (1995).

