# Supplementary material for: A Shh/Gli-driven three-node timer motif controls temporal identity and fate of neural stem cells
Source: Sci Adv. 2020 Sep 16;6(38):eaba8196. doi: 10.1126/sciadv.aba8196 (PMC7494341; doi:10.1126/sciadv.aba8196)
Supplement: aba8196_SM.pdf [file aba8196_SM.pdf]

Science Advances
AAAS

advances.sciencemag.org/cgi/content/full/6/38/eaba8196/DC1

Supplementary Materials for

A Shh/Gli-driven three-node timer motif controls temporal identity and fate of neural stem cells

José M. Dias, Zhanna Alekseenko, Ashwini Jeggari, Marcelo Boareto, Jannik Vollmer, Mariya Kozhevnikova, Hui Wang, Michael P. Matise, Andrey Alexeyenko, Dagmar Iber, Johan Ericson*

*Corresponding author. Email: johan.ericson@ki.se

Published 16 September 2020, *Sci. Adv.* **6**, eaba8196 (2020)
DOI: 10.1126/sciadv.aba8196

This PDF file includes:

Figs. S1 to S9
Table S1

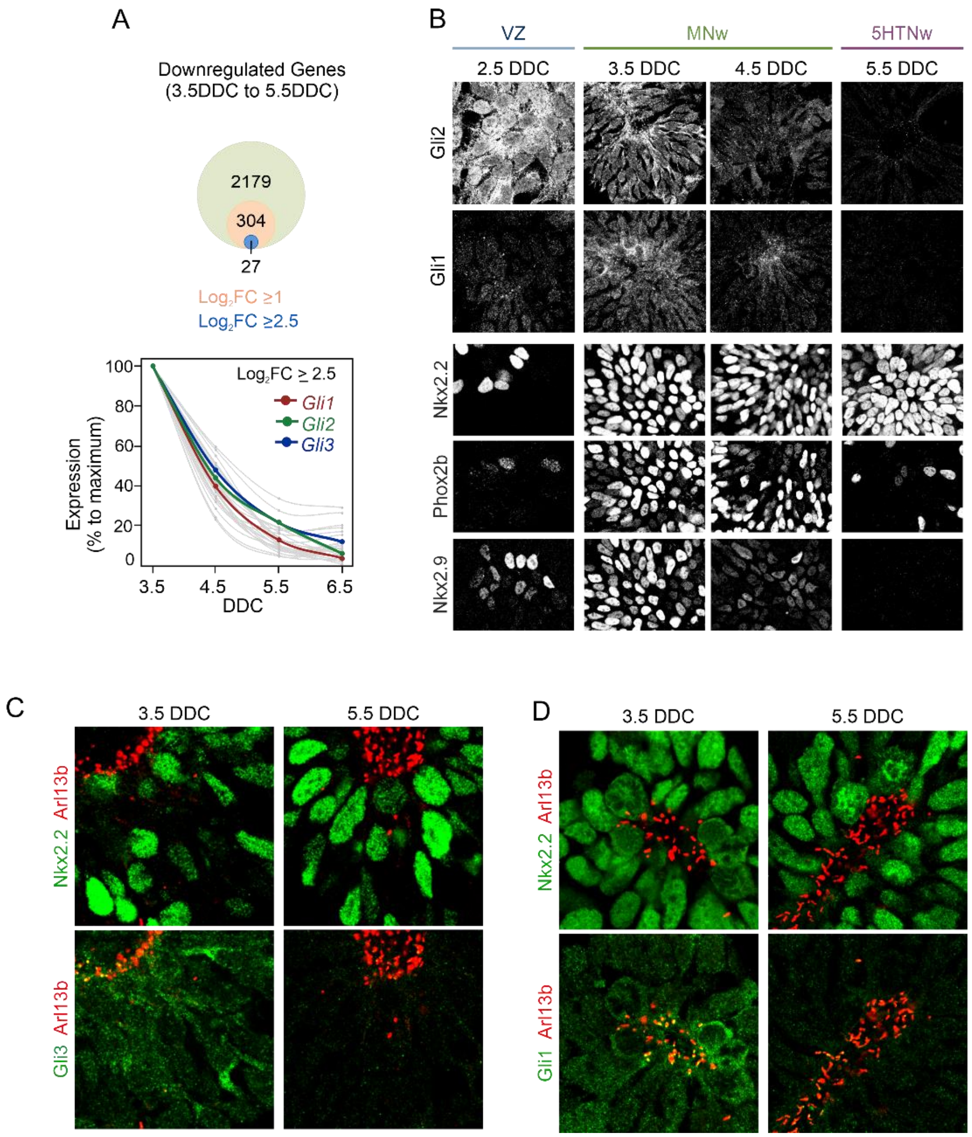

Fig. S1. Characterization of Gli expression in the temporal lineage. (A) Genes downregulated in NSCs between 3.5 and 5.5 DCC ($p \leq 0.05$) at different fold-change (FC) cut offs, and relative temporal expression profile of genes having a log2(FC)≥ 2.5. **(B)** Immunofluorescence of Gli1, Gli2, Nkx2.2, Phox2b and Nkx2.9 in neural progenitors at 2.5-5.5DDC. **(C, D)** Immunofluorescence of the cilia marker Arl13b with Gli3 (C) or Gli1 (D) in Nkx2.2^{+} progenitors. Images presented correspond to a triple immunocytochemistry with antibodies against Arl13b and Nkx2.2 with Gli3 (C) or with Gli1 (D), and where Arl13 immunostaining is presented with Nkx2.2 and with Gli3 or Gli1.

DDC, days in differentiation conditions.

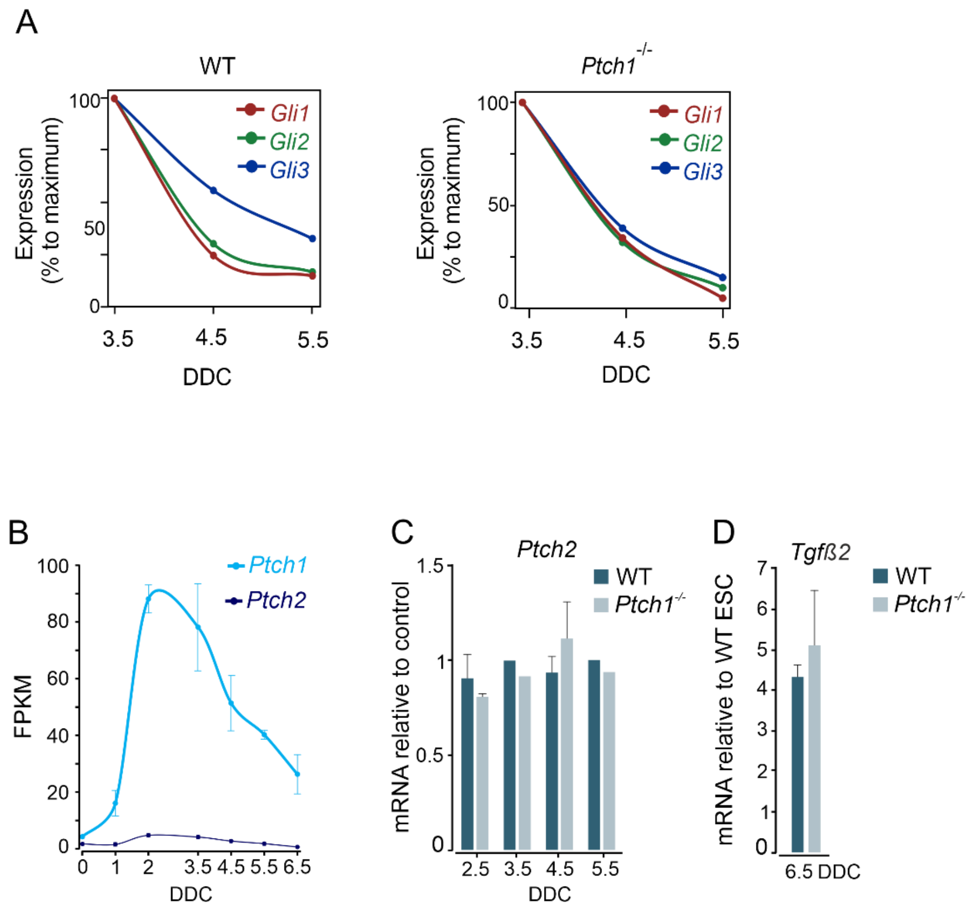

Fig. S2. Characterization of the expression of *Gli, Ptch* and *Tgfβ2* genes in *Ptch1*$^{-/-}$ cells.
(A) qPCR analysis of *Gli1,-2, -3* gene expression in WT and *Ptch1*$^{-/-}$ mutant neural progenitors isolated at 3.5, 4.5, and 5.5 DDC. **(B)** Expression levels, in FPKM, of *Ptch1* and *Ptch2* in WT neural progenitors isolated at different DDC defined by RNAseq. **(C, D)** qPCR analysis of *Ptch2* and *Tgfβ2* expression in WT and *Ptch1*$^{-/-}$ neural progenitors during differentiation. **(B-D)** Values, mean ± S.D.

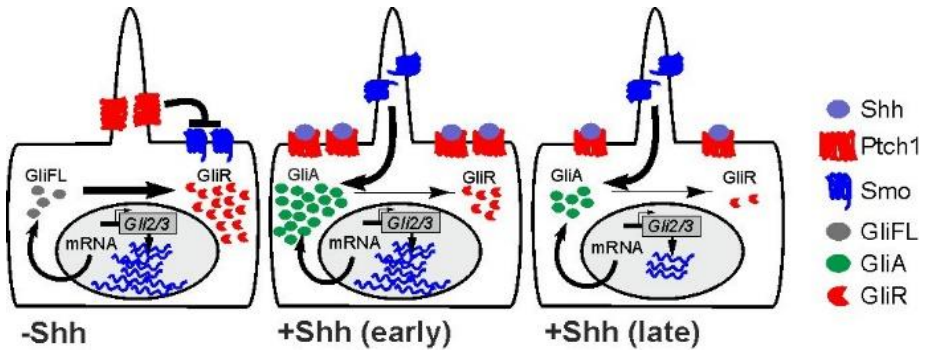

Fig. S3. Schematic summary of Gli dynamics in the temporal lineage. Bifunctional Gli proteins are processed into repressors in the absence of Shh. A proportion of Gli2 and Gli3 proteins are processed into GliR-forms even when Smo is fully activated, and GliA and GliR levels generated correlate to *Gli* transcription levels, which decrease over time.

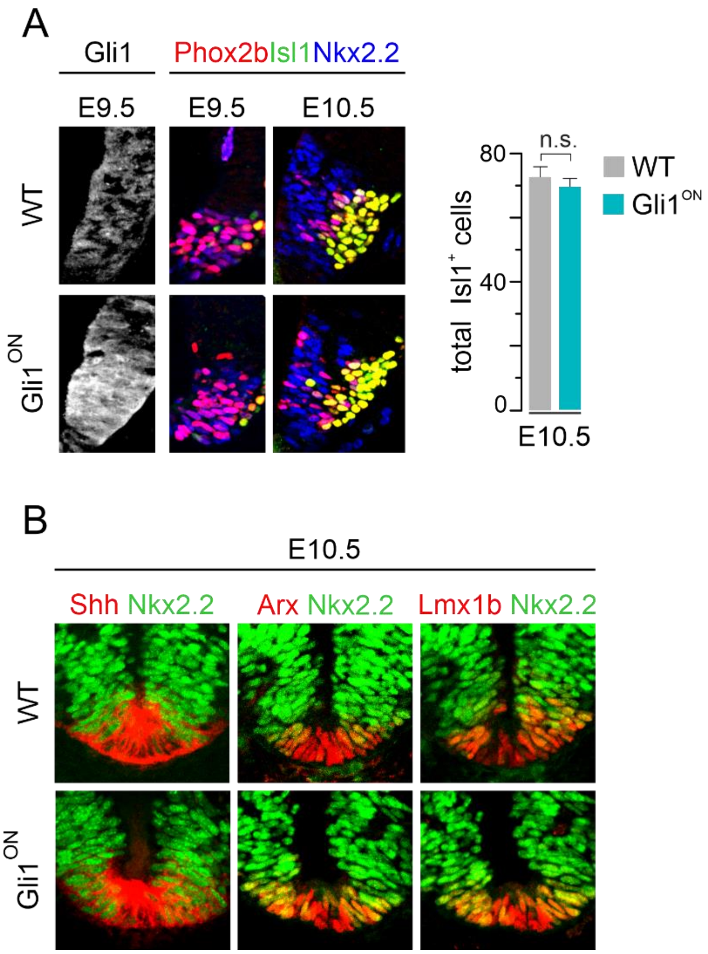

Fig. S4. Analysis of patterning and early MN production in Gli1^{ON} mutants.

(A) Immunostaining for Gli1, Phox2b, Isl1, and Nkx2.2 at rhombomere 7 of the hindbrain in WT and Gli1^{ON} embryos at E9.5 and E10.5, and quantification of Isl1^{+} MNs at E10.5. Values presented as mean ± S.D.. Student´s *t* test, n.s. non-significant. **(B)** Expression of the FP markers Shh, Arx, Lmx1b and of Nkx2.2 at caudal levels of the hindbrain in E10.5 WT and Gli1^{ON} embryos. Nkx2.2, which is transiently expressed in ventral midline cells at early developmental stages failed to be downregulated in the FP of Gli1^{ON} mice. However, this did not affect overall FP identity.

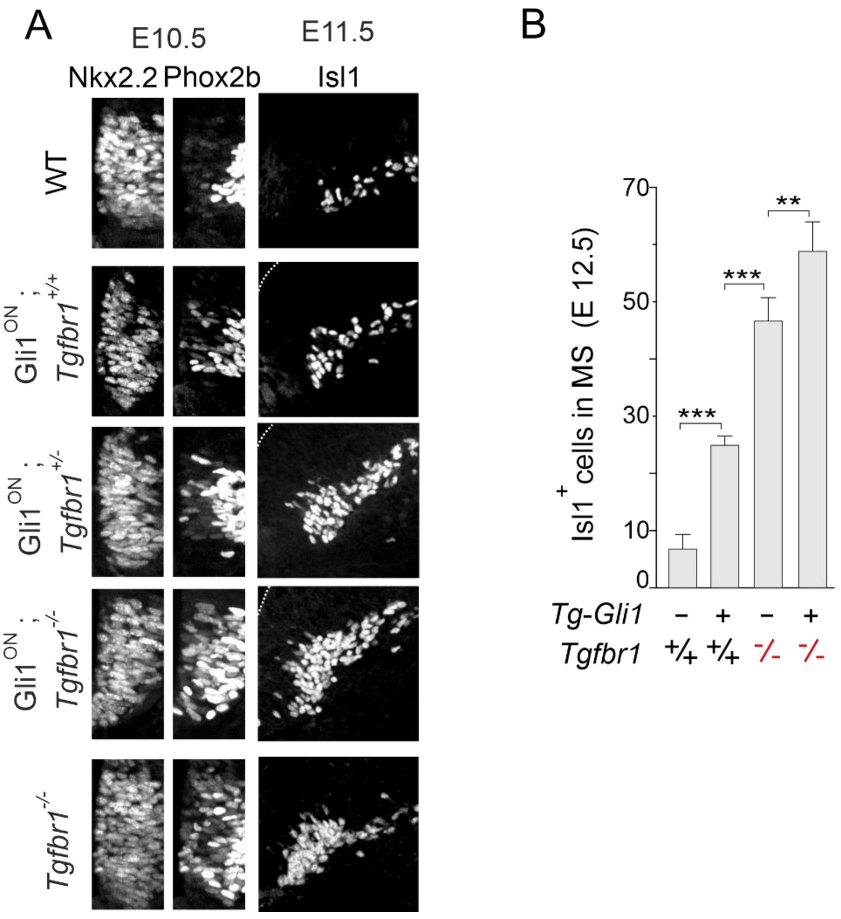

Fig. S5. Effects of reducing Tgfβ signaling in Gli1^{ON} mice on Phox2b expression in progenitors and motor-neuron production. (A) Transverse sections at r2/3 level of ventral hindbrain of mouse embryos at E10.5 and E11.5. Expression of Phox2b in Nkx2.2^{+} NSCs at E10.5 and Isl1^{+} motor neurons (MNs) located laterally to the Nkx2.2 progenitor domain at E11.5 in WT, Gli1^{ON}, *Tgfbr1*$^{-/-}$ embryos and Gli1^{ON} mutants on a *Tgfbr1* heterozygous (+/-) or homozygous (-/-) background. Isl1^{+} MNs at this level migrate laterally where they settle to form the trigeminal nuclei. Thus, Isl1^{+}-MN located in the trigeminal nuclei correspond to early-born MNs while those located laterally to the progenitor domain correspond to late-born MNs and can, therefore, be used as a measure of the temporal output of Nkx2.2^{+} progenitors. **(B)** Quantification of Isl1^{+} MNs in the migratory stream (MS) at E12.5 in WT (*n*=5), Gli1^{ON} (*n*=4), *Tgfbr1*$^{-/-}$ (*n*=5) and Gli1^{ON}: *Tgfbr1*$^{-/-}$ (*n*=3) embryos. Error bars, mean±S.D.; Asterisks, Student´s *t* test, ** $p \leq 0.01$, *** $p \leq 0.001$.

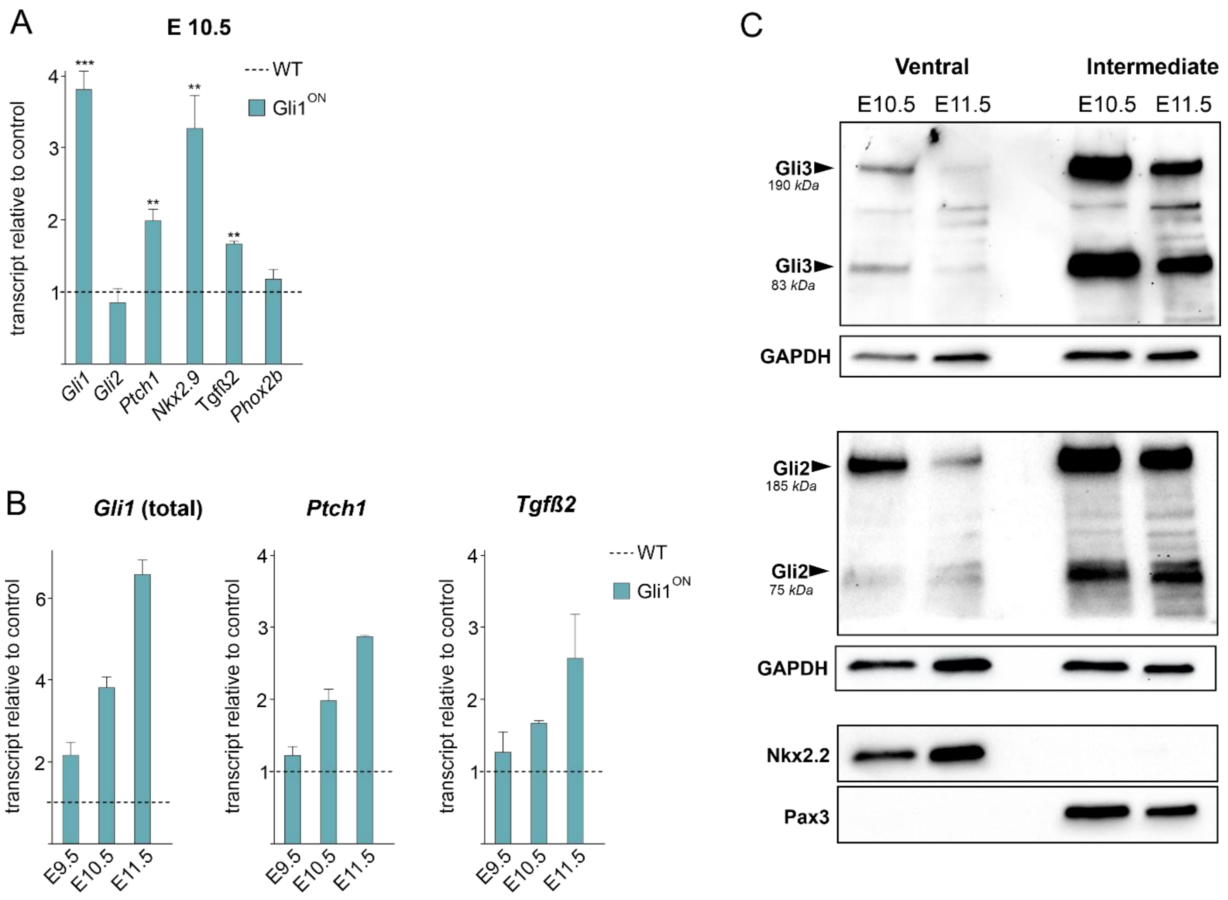

Fig. S6. Gene and protein expression analysis in hindbrain tissue over time.

(A, B) Gene expression analysis by qPCR on isolated ventral neural tissue from WT and Gli1^{ON} embryos at rhombomere 2-3 level of the hindbrain. Expression of *Gli1*, *Gli2*, *Ptch1*, *Nkx2.9*, *Tgfβ2* and *Phox2b* at E10.5 **(A)** and of *Gli1* (total), *Ptch1* and *Tgfβ2* at E9.5, E10.5 and E11.5 **(B)** in Gli1^{ON} embryos relative to WT embryos. *Gli1* (total) qPCR primers detect both endogenous and transgene *Gli1*. **(C)** Western blot analysis for Gli2, Gli3, Nkx2.2 and Pax3 proteins on micro-dissected ventral and intermediate hindbrain neural tissue isolated at E10.5 and E11.5. Nkx2.2 protein is expressed exclusively in ventral progenitors while Pax3 is expressed in intermediate/dorsal progenitors of the hindbrain, and were used as a control of tissue dissection.

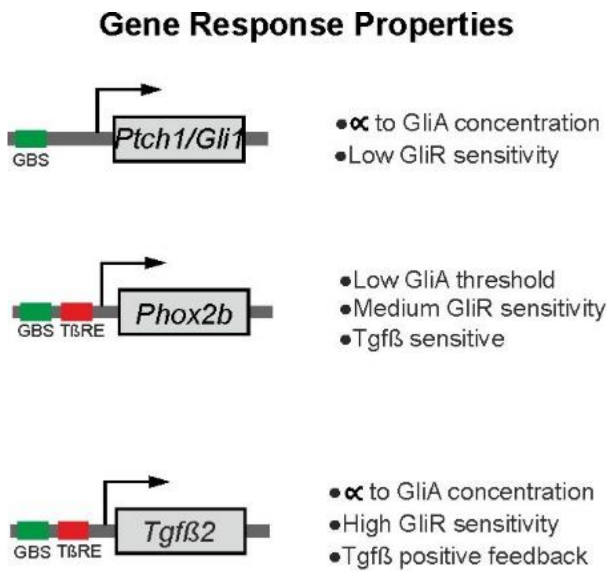

Fig. S7. Summary of gene response properties. Summary of gene response to GliA, GliR and Tgfβ signaling for *Ptch1*, *Gli1*, *Phox2b* and *Tgfβ2* genes.

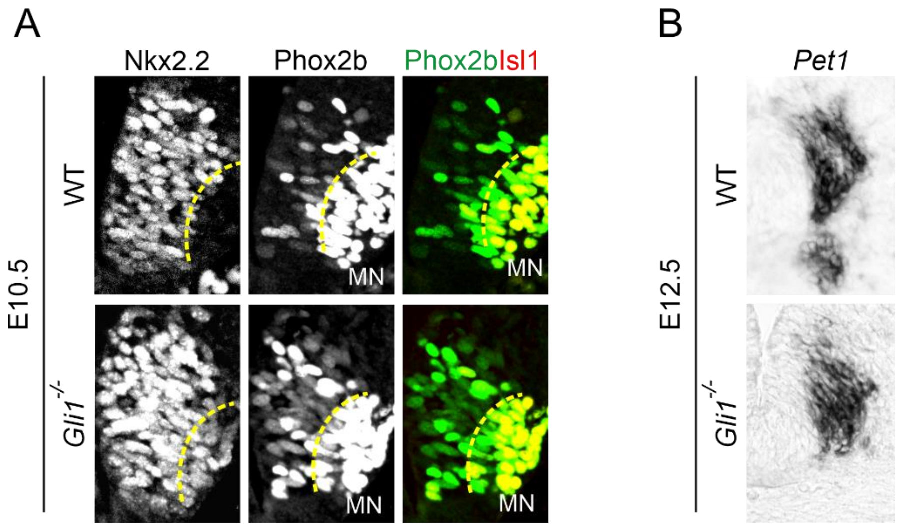

Fig. S8. Analysis of Phox2b expression in progenitors and 5HTN production in *Gli1*$^{-/-}$ embryos. (A) Immunofluorescence of Nkx2.2, Phox2b and Isl1 in WT and *Gli1*$^{-/-}$ embryos at E10.5. Isl1 identifies post-mitotic motor neurons (MN). **(B)** Expression of the 5HTN marker *Pet1* in WT and *Gli1*$^{-/-}$ embryos at E12.5 at r2/3 level of the hindbrain.

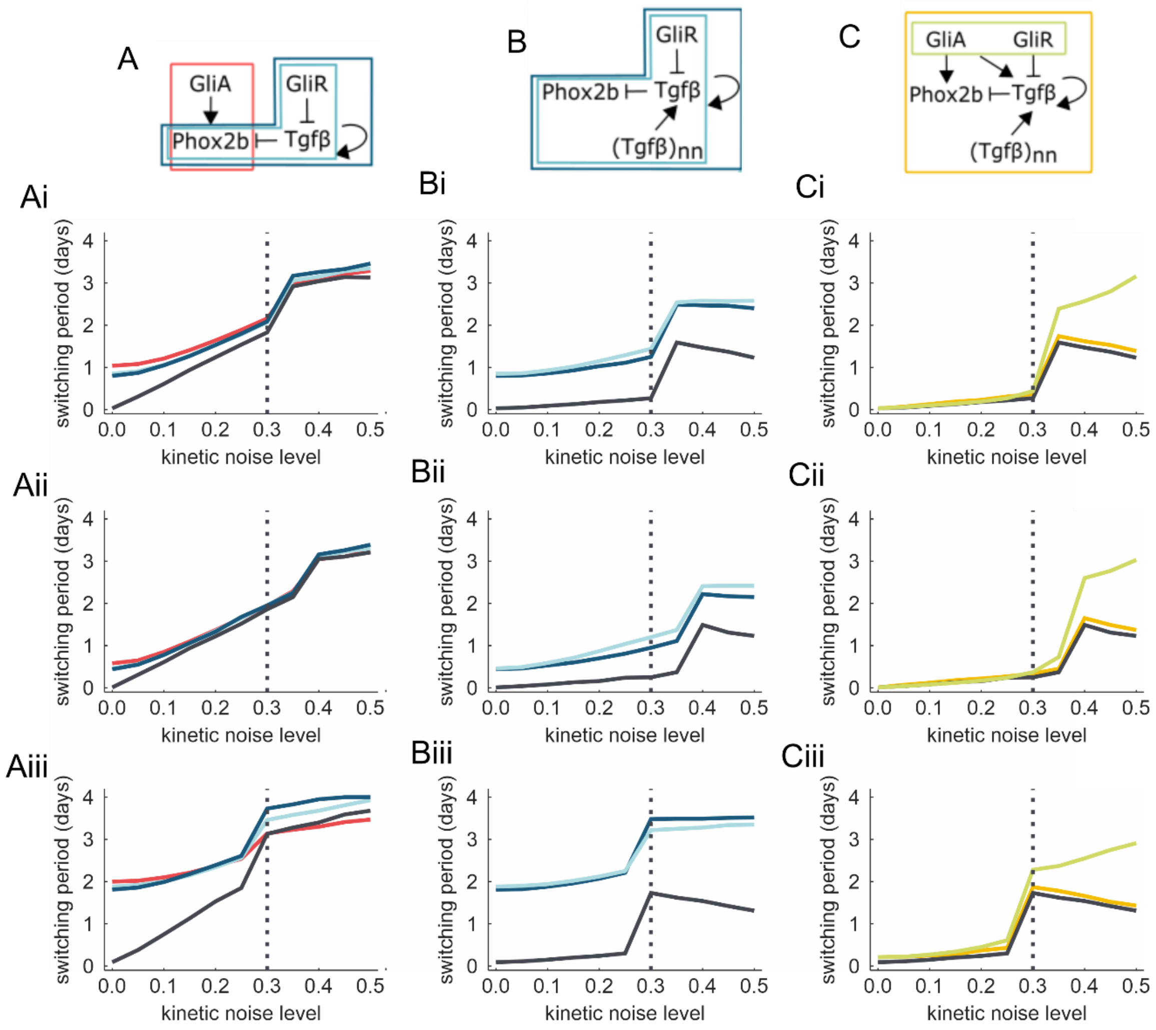

Fig. S9. Effects of different levels of threshold noise on the time interval to switch from MN-to-5HTN differentiation. Time interval to switch from MN-to-5HTN differentiation (in days) as a function of kinetic noise level for the regulatory networks defined in (A) (B) or (C) (see Fig.6) and considering different levels threshold noise: threshold noise used in Fig. 6G-I (Ai, Bi, Ci) or with a 3x lower (Aii, Bii, Cii) or 3xhigher (Aiii, Biii, Ciii) threshold noise.

Table S1. Biphasic and Cyc-repressed genes bound by Gli1 and Gli3 proteins

Biphasic & Cyc-repressed genes	Gli1+3-bound	Gli1-bound only	Gli3-bound only
Aco1	X		
Cxcr7	X		
Gli1	X		
Gpt2	X		
Grrp1	X		
Hhip	X		
Kcnc3	X		
Nkx2-9	X		
Nkx6-2	X		
Olig2	X		
Phox2b	X		
Ptch1	X		
Ptch2	X		
Rab34	X		
Vangl1	X		
9430020K01Rik		X	
Isl1		X	
Pdzd2		X	
Sox2ot		X	
Timp3		X	
Car14			X
Chrna7			X
Ddc			X
Doc2a			X
Dsp			X
Frat2			X
Heg1			X
Lhx4			X
Mapk11			X
Olig1			X
Pappa			X
Robo2			X
Sfrp1			X
Tecrl			X
Tns1			X
1810011O10Rik			
9130410C08Rik			
Angptl1			
Ano1			
Arid3b			
Armcx4			
Calcrl			
Dleu2			
Ebf2			
Erdr1			
Fabp5			
Fabp5l2			
Fbxo48			
Flt4			
Fndc3c1			
Hsd11b2			
Il4ra			
Lgi4			
Lhx3			
Litaf			
Mapk12			
Mmp17			
Neurod4			
Rasgef1c			
Sh3pxd2a			
Thsd4			
